# Microsaccade-rhythmic modulation of neural synchronization and coding within and across cortical areas V1 and V2

**DOI:** 10.1371/journal.pbio.2004132

**Published:** 2018-05-31

**Authors:** Eric Lowet, Bart Gips, Mark J. Roberts, Peter De Weerd, Ole Jensen, Jan van der Eerden

**Affiliations:** 1 Faculty of Psychology and Neuroscience, Maastricht University, Maastricht, the Netherlands; 2 Radboud University, Donders Institute for Brain, Cognition and Behaviour, Nijmegen, the Netherlands; 3 Maastricht Centre for Systems Biology (MaCSBio), Maastricht University, Maastricht, the Netherlands; 4 Centre for Human Brain Health, School of Psychology, University of Birmingham, Birmingham, United Kingdom; Massachusetts General Hospital, United States of America

## Abstract

Primates sample their visual environment actively through saccades and microsaccades (MSs). Saccadic eye movements not only modulate neural spike rates but might also affect temporal correlations (synchrony) among neurons. Neural synchrony plays a role in neural coding and modulates information transfer between cortical areas. The question arises of how eye movements shape neural synchrony within and across cortical areas and how it affects visual processing. Through local field recordings in macaque early visual cortex while monitoring eye position and through neural network simulations, we find 2 distinct synchrony regimes in early visual cortex that are embedded in a 3- to 4-Hz MS-related rhythm during visual fixation. In the period shortly after an MS (“transient period”), synchrony was high within and between cortical areas. In the subsequent period (“sustained period”), overall synchrony dropped and became selective to stimulus properties. Only mutually connected neurons with similar stimulus responses exhibited sustained narrow-band gamma synchrony (25–80 Hz), both within and across cortical areas. Recordings in macaque V1 and V2 matched the model predictions. Furthermore, our modeling provides predictions on how (micro)saccade-modulated gamma synchrony in V1 shapes V2 receptive fields (RFs). We suggest that the rhythmic alternation between synchronization regimes represents a basic repeating sampling strategy of the visual system.

## Author summary

During visual exploration, we continuously move our eyes in a quick, coordinated manner several times a second to scan our environment. These movements are called saccades. Even while we fixate on a visual object, we unconsciously execute small saccades that are termed microsaccades (MSs). Despite MSs being relatively small, they are suggested to be critical to maintain and support accurate perception during visual fixation. Here, we studied in macaques the influence of MSs on the synchronization of neural rhythms—which are important to regulate information flow in the brain—in areas of the cerebral cortex that are important for early processing of visual information, and we complemented the analysis with computational modeling. We found that synchronization properties shortly after an MS were distinct from synchronization in the later phase. Specifically, we found an early and spectrally broadband synchronization within and between visual cortices that was broadly tuned over the cortical space and stimulus properties. This was followed by narrow-band synchronization in the gamma range (25–80 Hz) that was spatially and stimulus specific. This suggests that the manner in which information is transmitted and integrated between early visual cortices depends on the timing relative to MSs. We illustrate this in a computational model showing that the receptive field (RF) of neurons in the secondary visual cortex are expected to be different depending on MS timing. Our results highlight the significance of MS timing for understanding cortical dynamics and suggest that the regulation of synchronization might be one mechanism by which MSs support visual perception.

## Introduction

Perception is an active process [1–3] in which animals explore their environment through specific movements of their sensory organs. For most animals that rely on visual perception, particularly for primates, large and small saccadic eye movements play a critical role in visual exploration [4–6]. The role of saccades in primate perception and cognition (e.g., visual attention) has been intensively studied over recent decades [4,5,7–9]. Even during fixation, gaze direction is not stable: the eyes continuously exhibit small saccadic movements (<1 degree). These small eye movements appear to be mainly controlled by the same neural circuitry as larger saccades [8,10–16]. These small saccades, termed microsaccades (MSs), have a marked impact on neural activity over the whole visual subcortical and cortical circuitry [5,13–15,17–22]. They modulate neural spike rates [7,8,16,19,20,22–26], spike bursting [20,21], and neural synchrony [15,25,27–31]. MSs may be important to refresh the visual image [32–34], for optimal local sampling during natural viewing [6,35–37], cognitive load [38], mental fatigue [39], and for visual attentional selection [8,9,14,40,41]. They have also been suggested to counteract fading/adaptation [37,42–46], in tandem with other fixational eye movements and saccades ([32,34,37] but see [47,48] for the alternative view). MSs, in tandem with drifts [26,49], have been shown to transform stationary spatial information into temporal modulations with important implications for visual coding [50,51].

All these demonstrations of the relevance of MSs for a variety of sensory and cognitive functions and of their effects on neural activity raise the question: how do MSs influence neural processing and cognition? Use of trial-averaging to improve signal-to-noise ratio (SNR) has for a long time obscured their role in the highly nonstationary neural processing of visual input [52]. Recent studies using single-trial analysis have shown that neural activity exhibits strong temporal variation locked to low-frequency rhythms [5,27,53–58]. Low-frequency rhythms (delta and theta frequencies, 0.5–8 Hz) have been shown to play a role in various sensory areas as well as in other subcortical and cortical areas [57–61]. In the primate visual cortex, low-frequency rhythmic activity in the delta/theta range correlates with the MS rhythm [27,28,53], indicating that MSs are associated with important timescales of neural variation and cognition [8,25,50,62–66].

These findings may relate to other studies demonstrating that MSs can enhance synchronization-based neural coding [20,33,67,68], in particular during MS-induced transients [20,21]. The “reset” of neural activity accompanying these transients [27,69] may enable latency coding [20,70] for fast and efficient information transfer of new visual input. Indeed, it has been shown that the first spike after a saccade is highly informative of the stimulus [71]. However, neural synchronization occurs not only at the transient shortly after the MS but also throughout the interval between MSs in the form of longer-lasting narrow-band oscillations [27,53,69,72]. How neural synchronization is organized by the MS rhythm and how it affects neural coding are not well understood.

In the current work, we aimed to investigate the transient activity directly following an MS and contrast this with the later “sustained” activity that lasts until the next MS. We showed differences in synchronization and coding properties during these 2 saccade-locked time intervals, through computational modeling and local field potential (LFP) recordings in macaque early visual cortex. We used spatial excitatory–inhibitory spiking network model receiving MS-modulated input that was constrained by previously reported spectral dynamics of monkey V1 and V2 LFPs [53]. We found that synchronization properties within and across cortical areas differed between the transient and the sustained period.

During the transient response, synchrony at various frequency bands was high, regardless of the stimulus pattern that we presented to the network. In contrast, in the sustained period, synchronization in the gamma range became highly selective, spatially local, and shaped by both the underlying connectivity as well as stimulus properties, within V1 and between V1 and V2. Using our model, we predict that the different synchronization properties within an MS interval have implications for the shaping of the downstream receptive fields (RFs) as well as impact on the effectiveness of different neural coding schemes. We conclude that the early, highly synchronized, transient activity that immediately follows an MS permits rapid initial coding of the visual input. On the other hand, the following sustained activity with local synchrony allows context-dependent coding. This 2-step MS-linked processing can likely be generalized to large saccades [7,18] and possibly to other rhythmic sensory sampling processes as well, such as sniffing [2,73] and whisker movement [1,65,74].

## Results

### Modeling of MS-induced V1 neural oscillatory dynamics

**Fig 1:**
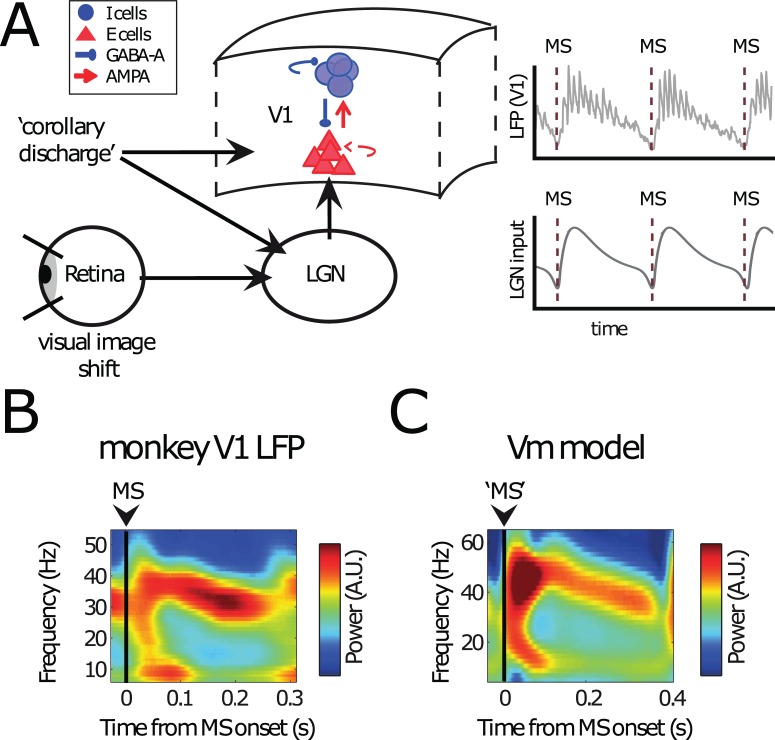
Modeling MS V1 neural dynamics. (A) Conceptual overview over the model. A (small) region in V1 was modeled as a PING network of spiking excitatory (E cells, regular-spiking) and inhibitory neurons (I cells, fast-spiking). They were connected through AMPA- and GABA-A-type synapses (see Materials and methods and Fig 2A for details). On the PING network, we imposed currents mimicking MS-modulated input from LGN and/or corollary discharges to V1 (see Materials and methods). We used the spikes and approximate LFPs in the PING network for subsequent analysis. (B) A representative experimental MS-triggered TFR of V1 LFP power. MSs occurred at *t* = 0 s. Note the broadband activity just after the saccade onset (0–100 ms) followed by a narrow-band gamma signal (100 ms onwards). (C) An MS-triggered TFR of the simulated LFP. Conventions as in panel B. The Vm is able to produce both a broadband signal directly following the saccade onset (transient) as well as the narrow-band gamma response afterwards (sustained). AMPA, α-amino-3-hydroxy-5-methyl-4-isoxazolepropionic acid; GABA, gamma-aminobutyric acid; LFP, local field potential; LGN, lateral geniculate nucleus; MS, microsaccade; PING, Pyramidal-InterNeuron Gamma; TFR, time-frequency representation; Vm, model visual cortex.

We constructed a model network (Fig 1A) to study the effects of MSs. At the core of our model is an excitatory–inhibitory neural network mimicking V1 (note that the neurons are not explicitly made orientation selective or show “on” or “off” regions; for details, see Materials and methods). The connections between the excitatory and inhibitory cells made it possible for the network to produce Pyramidal-InterNeuron Gamma (PING) rhythms [75,76]. In our model, V1 received input from lateral geniculate nucleus (LGN) relay neurons, here represented by a direct current input. For the saccadic modulation of the spike rate of the LGN relay neurons, we followed the experimental results of [7,17], using a double exponential kernel (see Materials and methods and bottom-right plot in Fig 1A). Note that it is not completely clear whether the modulations in the LGN firing rate are due to image shifts on the retina, corollary discharges, or both [15]. Our model does not attempt to distinguish between these 2 possible contributing factors. Furthermore, the input pattern to the network are fixed, and the MS-induced modulations were added, which means that input patterns did not change with each MS. This had the advantage that we were able to compute the oscillatory and synchronization characteristics reliably for a given input pattern. Control analysis with changing input patterns are shown in the S1 Text.

Fig 1B shows a representative MS-onset–triggered time-frequency representation (TFR) of the LFP recorded in monkey V1 [27,53]. Shortly after the MS, we observed a power increase in a frequency range covering alpha/beta frequencies shortly after the MS as well as broadband gamma. It has been shown that the spectral changes are linked to the MS-evoked response [27,53] and are associated with spectral phase reset in the alpha/beta frequency range [27]. Broadband gamma after MS or saccades has also been previously reported [27,28,77]. The spectral response resembled the transient responses after stimulus onset described by [78]. Because the image shift on the retina induced by the saccade physically resembles a stimulus onset, it is likely that it represents the main drive for this transient response. However, corollary discharge and ongoing rhythmic activity can further shape the transient response.

Around 100 ms after the MS, gamma activity was observed in a frequency band that was narrower and lower than before (25–50 Hz). This gamma activity remained until the next MS. Alpha/Beta power was reduced during this period. This period resembles the spectral profile described for the “sustained” response following stimulus onset [78]. Strictly speaking, the observed dynamics of gamma rhythms cannot be termed “sustained” or “stable” because gamma synchrony is short-lived and the frequency decreases over time. However, to contrast with the strong transient dynamics in the early period after the MS, we keep the name “sustained” for simplicity.

**Fig 2:**
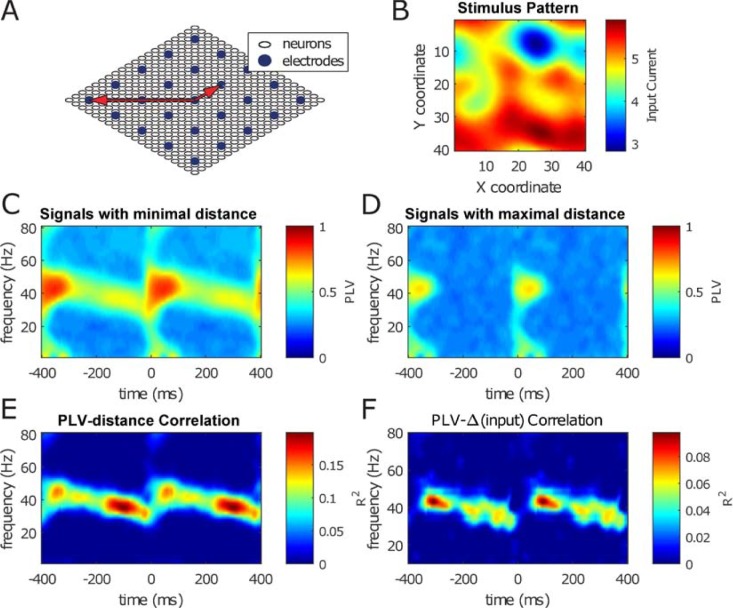
Synchrony across the network depends on connection strength and input difference more strongly during sustained gamma-band activity than during MS-induced transients. Synchrony was measured by PLV. (A) A scaled-down schematic representation of the model network. Inhibitory neurons are omitted for clarity. The full network consists of excitatory neurons placed on a square 40 × 40 grid together with a square grid of the same diameter containing 20 × 20 inhibitory neurons (not depicted). LFP electrodes are placed on a 10 × 10 grid spread equally across the 40 × 40 neuronal grid. The boundaries of the grid are periodic, i.e., the neurons are placed atop a toroidal surface. The 2 red arrows indicate 2 electrode pairs: a neighbor pair, at minimal distance, and a pair at maximal distance. (B) Network was driven by input consisting of retinotopically smoothed Gaussian white noise. This input current was modulated over time by multiplying it by the MS modulation kernel (see Fig 1A, below right) for a total of 50 MSs. The SNR factor of the temporal Gaussian noise was equal to 2 (see Materials and methods). (C) Mean synchrony strength expressed as the mean PLV (see Materials and methods) across MSs between neighboring LFP electrodes (see smallest of the red arrows in panel A). (D) Same as panel C, but now between LFP electrodes with maximal distance to each other (see longest of the red arrows in panel A). Note the lack of synchrony in the gamma band during the sustained period (t = 150–350 ms). (E) The correlation coefficients between electrode distance and PLV for a broad range of frequencies and all intersaccade time points. Only the synchrony in the narrow-band gamma activity (25–50 Hz) during the sustained period depends on the electrode distance. (F) The same as panel E but for stimulus input differences. Only the neuron pairs that are within 4 interneuron distances from each other were used for generating this figure. Neurons further apart have little to no synchrony during the sustained period (i.e., the correlation in panel E is negative) and were therefore not included. LFP, local field potential; MS, microsaccade; PLV, phase-locking value; SNR, signal-to-noise ratio.

In Fig 1C, we show the average TFR of power calculated for the network simulations (for details on the network, see Fig 2) triggered by the MS. The network spectral dynamics were similar to the observed V1 spectral dynamics with early transient alpha/beta as well as broadband gamma and later, more sustained narrow-band gamma power. Despite spiking being irregular, MSs led to a short, strong alignment of many excitatory neurons, which led, in turn, to power in the lower frequency bands (<20 Hz). The gamma oscillations, emerging from excitatory–inhibitory interactions, also exhibited behavior similar to the V1 experimental data, including decreasing frequency over time. The decrease of gamma frequency is prominent when looking on linger MS intervals (S1 Fig and S2 Fig). It has been shown that the dominating gamma frequency is dependent on input strength [75,79–83]. Therefore, the observed pattern of a gamma frequency that decreases over time can be attributed to the exponential decay in the MS spike rate modulation function (Fig 1A). In addition, adaptation properties of pyramidal cells could also play a role [5,32], but we did not explicitly investigate this question. Overall, the network simulations yielded a satisfying replication of the spectral dynamics observed in monkey V1 in response to saccades.

### Within-area cortical synchronization properties are modulated by the MS rhythm

The main goal of this study was to investigate the information processing properties during the “transient” and “sustained” period after an MS. In Fig 2, we illustrate that gamma synchrony in the sustained period depends on distance within the network, whereas this is not the case in the transient period. To arrive at that conclusion, we used further simulations of the visual network model used to generate Fig 1C.

The structure of the network is shown in Fig 2A. The network consisted of a 40 × 40 grid of excitatory regular-spiking cells (RSs) overlaid by a 20 × 20 grid of inhibitory fast-spiking cells (FSs). The connectivity within the network decreased with distance according to Gaussian connection probabilities. Periodic boundary conditions were applied to decrease finite size effects. A 10 × 10 grid of virtual electrodes was positioned over the grid that averaged the local membrane potentials according to a Gaussian kernel to estimate an LFP. For more details on the network structure, see Materials and methods.

The retinotopic input to the RS cells before applying MS modulation is shown in Fig 2B. This input consisted of a spatially low-pass filtered white noise pattern that was kept constant across simulated saccades. The phase-locking value (PLV; see Materials and methods) across the 50 simulated saccades was calculated for all pairs of LFP electrodes. The average PLVs for all neighboring electrodes are shown in Fig 2C, whereas the average PLVs for all pairs of neurons at maximal distance (keeping in mind the periodic boundary conditions of the network; see Fig 2A) are shown in Fig 2D. A striking difference between neighboring electrodes (panel C) and distant electrodes (panel D) is the lack of gamma-band (25–40 Hz) synchrony in the latter during the sustained period.

To further analyze this difference, we plotted the PLVs for gamma at 30 ms and at 300 ms post saccade as a function of electrode distance in Fig 2E (see the 2 white crosses surrounded by grey and black circles, respectively, in panels C, D, and E). A comparison of Fig 2C (nearby probes) and D (distant probes) shows similar magnitudes of early “transient” synchronization (at 30 ms) but very different magnitudes of later “sustained” synchronization (at 300 ms). This shows a striking distance dependence for the sustained but not the transient gamma synchronization. The distance dependence of the PLV is illustrated further in Fig 2E. Here, we plotted the correlation between PLV and distance across probes over all pairs as a function of time relative to MS onset. Panel E shows that only the gamma-band synchronization in the sustained period showed a significant linear correlation with cortical distance (red regions in time-frequency plot). Note that both connectivity strength and stimulus correlation dropped with distance, therefore both possibly contributed to the decline of sustained gamma synchronization with distance. In summary, gamma synchrony was higher between nearby neurons than between distant neuron pairs. This was true only for the band-limited gamma during the sustained phase, not during the transient. The distance dependency of sustained band-limited gamma remained for longer intersaccade intervals (S1 Fig) or when the input pattern to the simulated network was varied for different MSs (S3 Fig). We then tested whether synchronization reflects stimulus information (Fig 2F). Specifically, we investigated whether the amount of synchronization is related to the magnitude of stimulus input difference between model LFP contacts by running the network for different stimulus configurations. Similar to connectivity strength, we computed the linear correlation over all probe pairs between PLV value for a given frequency and time with the amount of input difference. We observed that stimulus variation was reflected specifically in the narrow-band gamma in the sustained phase, but not in the transient phase.

### Selective network synchronization only in the sustained period after MS

**Fig 3:**
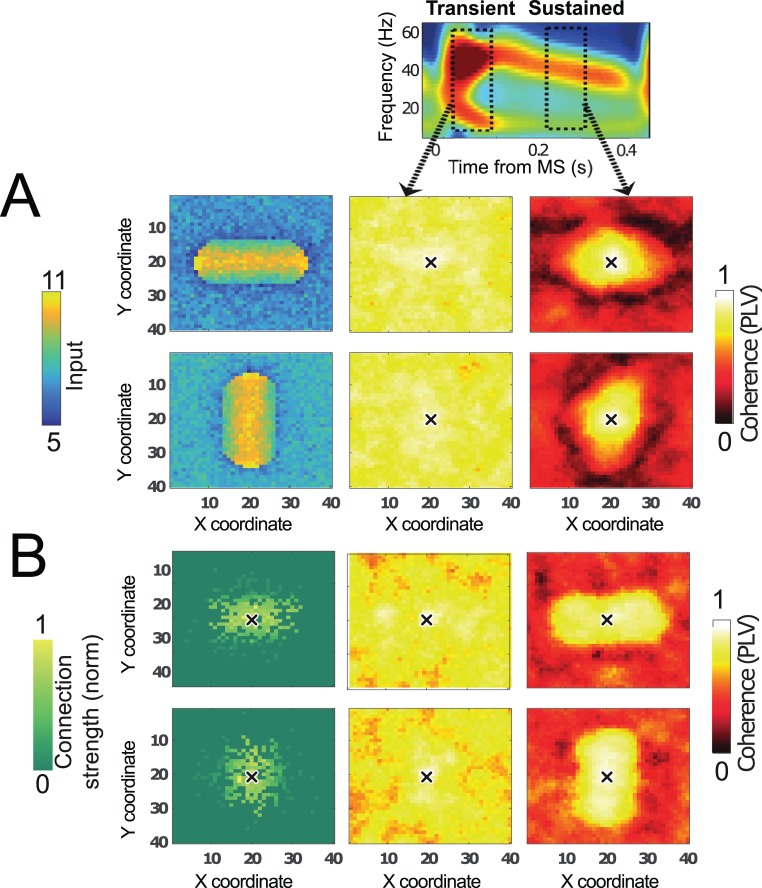
MS-dependent spatiotemporal organization of network activity. Network was a lattice with excitatory–inhibitory neurons locally connected with periodic boundary conditions (similar to Fig 2A). (A) Oriented stimulus, isotropic connectivity. Left: firing rates of excitatory RS neurons in the network that was driven by a horizontal (top) or vertical (bottom) bar-shaped stimulus. Network synchronization (PLV with the center neuron, denoted by a black cross, as reference) is shown in the transient (middle) and in the sustained (right) period with 50-ms time windows. Middle: in the transient period, PLV with the center neuron was high over the whole network due to the MS-induced reset. Right: in contrast, in the sustained period, the PLV was high only close to the reference neuron in the network center, and the PLV profile was shaped by the orientation of the bar-shaped stimulus. (B) Isotropic stimulus, anisotropic connectivity. Left: the center neuron (indicated by a black cross) was preferentially connected to neurons left and right from it (top), or to neurons below and above it (bottom). Middle: in the transient period throughout the network, the PLV with the center neuron (indicated by a black cross) was high. Right: during the sustained period, PLV was only high close to the reference neuron, and the shape of the PLV profile reflected the preferred connectivity orientation. MS, microsaccade; PLV, phase-locking value; RS, regular-spiking neuron.

As an illustration, we first manipulated input differences by giving the visual network model (which has isotropic local connectivity; see Fig 2A) 2 different spatial input driving patterns. In Fig 3A, a smoothed rectangular-shaped stimulus was presented to the network (left). To illustrate the effects on the gamma-band phase coordination in the transient and sustained period, we computed the spatial distribution of PLVs [84] referenced to the neuron in the center of the two-dimensional (2D) network (crosses in Fig 3A and 3B). The PLVs in the transient period were uniformly high (Fig 3A, middle). This is caused by the steep rise of MS-modulated input (Fig 1A), which is similar for all neurons in the network. During the sustained period, the distribution of PLVs was more local and reflected the orientation of the rectangular-shaped stimulus more closely (Fig 3A, right).

To illustrate the effect of connectivity on the PLV, we performed another simulation in which we altered the connectivity while keeping the input uniform across the network. We introduced horizontal or vertical anisotropy in the connectivity profile by altering connection probability of the model network (Fig 3B, left). The PLV distribution in the transient period was again high and uniform (Fig 3B, middle), whereas in the sustained period, it was local and dictated by the connectivity structure (Fig 3B right).

Taken together, Fig 3 supports the view that synchronization in the transient period is fundamentally different than in the later sustained period, which suggests that phase coordination in these periods relies on different mechanisms.

To formally test whether there are 2 phase-coordination mechanisms in play, we investigated whether the Arnold tongue [85] (see S1 Text) could be retrieved from the early (transient) and late (sustained) period after the MS. The Arnold tongue represents a triangular-shaped synchronization region in the 2D space of detuning (related to input difference) and interaction strength (related to connectivity strength). An Arnold tongue is expected if synchronization arises through locally mutually weakly interacting oscillators (see S1 Text and S4 Fig), a framework proposed for neocortical gamma synchronization [29,86–88]. We found an Arnold tongue only in the sustained part of the MS interval (S5 Fig).

### Replication of model results in monkey V1 cortical data

**Fig 4:**
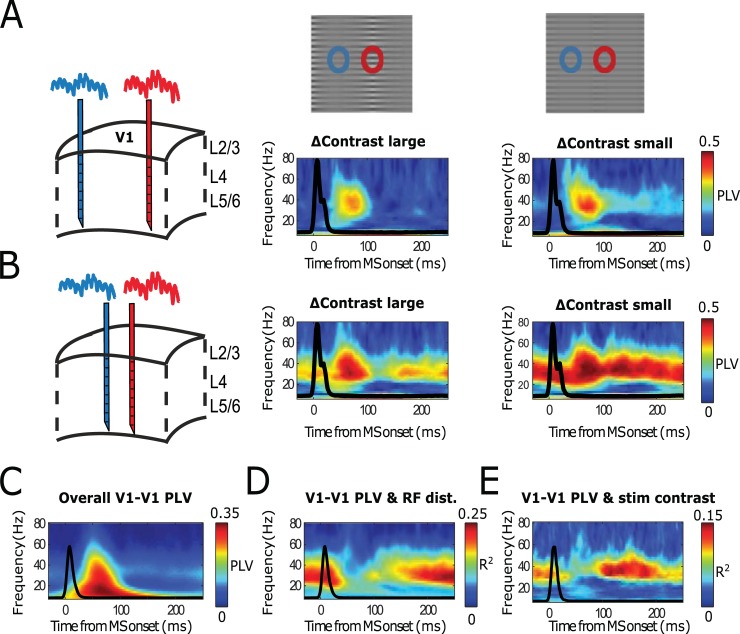
Monkey LFP measurements across V1 cortical locations replicate model results. (A–B) Single contact pair examples. (C–E) Population-level analysis (12 recording sessions, each with 3 laminar probes from 2 monkeys). (A) Temporally resolved PLVs for multiple frequencies between 2 LFP electrodes in V1 relatively far apart (4–6 mm,) as a function of time, aligned to the onset of MSs. Middle subplots represents PLV values for stimulus grating condition including large contrast variation. Right subplot represents PLV values with stimulus grating containing low contrast variation. Black lines represent MS-triggered averaged eye speed. (B) The same as panel A, but for relatively close probes (2–3 mm). (C) The population-averaged V1-V1 MS-triggered PLV spectrum. (D) Represents the explained variance of MS-triggered PLV values as a function of RF distance between probes. (E) Same as panel D, but as a function of stimulus contrast difference. LFP, local field potential; MS, microsaccade; PLV, phase-locking value; RF, receptive field.

To test the different synchronization properties in the transient and sustained time periods predicted from our simulations, we applied the analysis of the model results (Fig 2 and Fig 3) to electrophysiological recordings in monkey V1 (see Materials and methods). Fig 4 shows the results of this analysis. We collected LFPs from 3 simultaneously inserted laminar probes in macaque V1 (separated each by about 2–3 mm) while presenting the monkey with static whole-field gratings having spatial-varying contrasts [29]. For different stimulation conditions, the RFs of recorded cortical locations experienced different contrasts, known to modulate V1 neural activity [89]. The monkey received a reward when successfully keeping gaze on the fixation point during the whole trial. The eye position was monitored through an infrared camera system (see Materials and methods). We computed the current source density (CSD) along the laminar probe to get more local signals. In Fig 4A and 4B, we show example MS-triggered phase-locking spectra from single contact pairs from probes in different V1 cortical locations. In Fig 4A, the distance between probes was relatively large (around 5 mm), whereas in Fig 4B, distance was shorter (around 2 mm). For these examples, we show PLV spectra for stimulus conditions with large (left) and low (right) contrast difference. As in the model, we observed strong transient synchronization irrespective of stimulus difference or cortical distance across the probes. Only the later, sustained synchronization was sensitive to the distance among probes (compare Fig 4A and 4B) and to the stimulus difference (compare within B middle and right-hand panels). In Fig 4C–4E, the population level confirmed that the narrow-band gamma in the sustained phase was specifically sensitive to the distance between probes and to stimulus differences, despite that in the averaged PLV spectra (Fig 4C), the transient broadband component dominated.

As in our modeling data, we tested whether we could reconstruct the Arnold tongue (triangular-shaped synchronization region in 2D space of detuning and interaction strength) in the transient and sustained period of the MS interval. The Arnold tongue is a hallmark of weakly coupled oscillator synchronization, which has been proposed to underlie neocortical gamma-band synchronization [29,90–92]. Similarly, we found that the Arnold tongue could only be reconstructed from the sustained gamma-band synchrony and not for transient synchrony (S6 Fig, see S1 Text for more details). This suggests that neural synchronization in the sustained part of the MS intervals shows dependence on input difference and interaction strength as expected from weakly coupled oscillators.

### V1-V2 cortical synchronization properties are modulated by the MS rhythm

**Fig 5:**
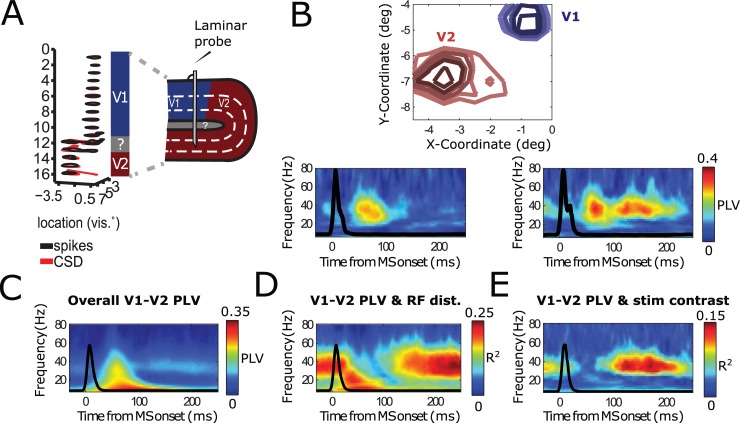
Monkey V1 and V2 cortical locations replicating model results. (A) Schematic illustration of how the laminar probes were inserted in V1 and reaching, in many cases, V2 lying beneath. Panel is taken from [29]. (B) Single contact pair examples. (C–E) Population-level analysis (12 recording sessions, each with 3 laminar probes from 2 monkeys). (B) Temporally resolved PLVs for multiple frequencies a contact pair situated in V1 and in V2, aligned to the onset of MSs. Upper panel shows the RFs of the corresponding. Lower subplots represent PLVs for stimulus grating condition including large (left) and large (right) contrast variation. (C) The population-averaged V1-V1 MS-triggered PLV spectrum. (D) Represents the explained variance of MS-triggered PLVs as a function of RF distance between probes. (E) Same as panel D, but as a function of stimulus contrast difference. MS, microsaccade; PLV, phase-locking value; RF, receptive field.

During our laminar recordings, we had frequent access to V2 neurons because the probes often extended beyond V1 deep layer, reaching into the deep-middle layers of V2 situated beneath (Fig 5A). We observed striking shifts in the RF position when contacts reached V2, where they were also clearly larger (for details see [29]). We did the same MS-triggered analysis for V1-V2 contact pairs (Fig 5). In Fig 5B, we depict an example V1-V2 pair. We observed similar MS-triggered PLV spectra, in which the narrow-band gamma rhythm in the sustain phase was particularly dependent on stimulus contrast differences. Applying the same population-level analysis as in Fig 4, we found that V1-V2 gamma-band synchronization in the sustained phase within the MS interval was informative about the RF distance between V1 and V2 contacts and the stimulus contrast experienced—even if, in the overall MS-triggered PLV spectra, the broadband-transient PLV component with dominant lower frequencies was more striking.

**Fig 6:**
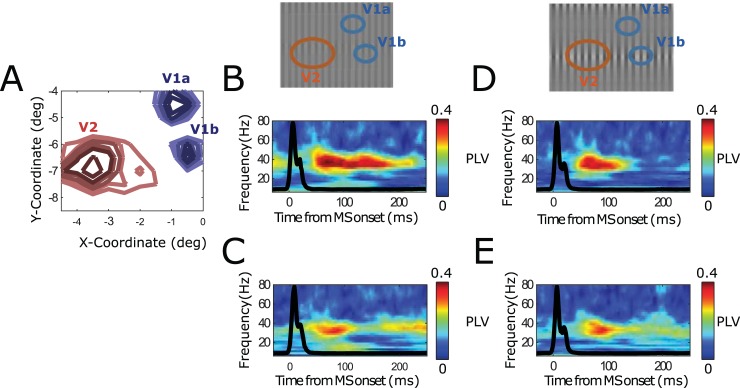
Illustration of differential synchronization of 2 V1 locations to 1 V2 location (from 1 session, Monkey M1). (A) The RF locations of the 2 V1 locations (V1a, V1b) and V2 location. (B–C) Stimulus grating had uniform contrast. (B) V1a-V2 MS-triggered TFR PLV. Squared dashed box indicates the sustained period. (C) Same as panel B, but for V1b-V2 MS-triggered TFR PLV. (D–E) Stimulus had varying spatial contrast. (D) V1a-V2 MS-triggered TFR PLV. (E) Same as panel D, but for V1b-V2 MS-triggered TFR PLV. MS, microsaccade; PLV, phase-locking value; RF, receptive field; TFR, time-frequency representation.

The different contribution of V1-V2 MS-triggered spectral locking as a function of stimulus properties means that, for a given stimulus, the synchronization of a given V2 location to V1 locations will change. In other words, the preferred integration of V1 neural space by V2 depends on the stimulus as well as the time relative to last MS time. To illustrate the differential contribution of V1 locations to V2 in terms of synchronization for different stimuli, we depicted in Fig 6 the MS-triggered synchronization between 1 V2 location and 2 V1 locations recorded simultaneously.

Our experimental data, in agreement with our model predictions, show in summary that synchronization within and across visual cortical areas showed marked differences as a function of the time window taken in relation to MS occurrence. Neural synchronization is an important mechanism to regulate and route information transfer between neural populations [93,94]. We therefore studied in the following segments the implications of MS-dependent synchronization changes for V1-V2 information transfer by extending our modeling framework.

### Implications of the transient and sustained mode of synchronization for information transfer between V1 and V2

**Fig 7:**
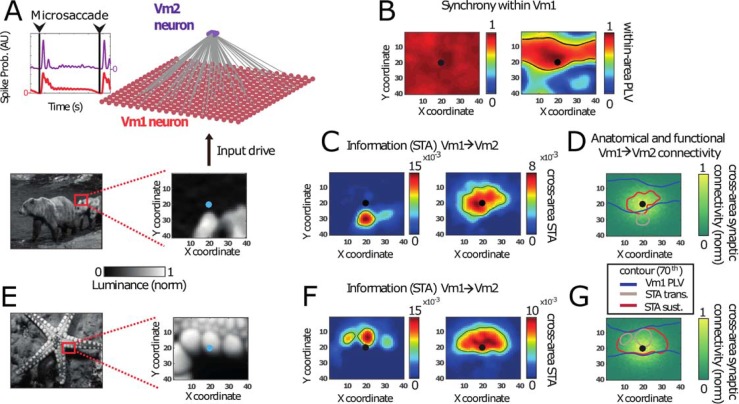
MS-dependent synchronization shapes information transfer to downstream neurons. (A) Excitatory–inhibitory visual model network, receiving MS-modulated spatially structured input derived from natural image luminance (bottom). The V1 network was unidirectionally connected to a downstream V2 neuron. (B) The synchronization profile (measured by PLV) of the center V1 neuron to all other neurons in V1 is shown for the input in panel A. During the transient period, synchrony was high across the V1 network, whereas synchrony was local and anisotropic in the sustained period. Synchrony was high for neurons receiving similar input. (C) Information transfer of V1 neurons to V2 (quantified as STA(V1→V2), see Materials and methods). (Left) In the transient period, _syn_STA was high for neurons with strong input (and therefore high firing rate). (Right) In contrast, in the sustained period, the _syn_STA map showed high values for neurons with similar input. (D) Contour lines of PLV in V1 during the sustained period (see panel B) as well as for the _syn_STA from V1 to V2 (see panel C) overlaid on of the anatomical connectivity. The _syn_STA maps (green and red contour lines) did not strictly follow the synaptic connectivity profile (heat map). During the transient, they were biased towards higher input. In the sustained period, they were biased towards higher synchrony (PLV, blue contours). (E) The same simulation as in panel A, but now using a different stimulus image, where the center neuron in V1 is situated over a bright patch. (F) Similar to panel C, but for the simulation using the stimulus in panel E. This second stimulus led to _syn_STA maps that were similar in both the transient (left) and sustained (right) periods. (G) Similar to panel D, but using the stimulus in panel E. With this stimulus, the _syn_STA maps showed large amount of overlap in both the transient and sustained periods. This is due to the fact that the neurons were synchronizing to the center neuron, which were also the neurons with high input. MS, microsaccade; PLV, phase-locking value; _syn_STA, synaptically confined spike-triggered average.

The different properties of synchronization within a visual area during the transient and sustained period after an MS should affect information transfer among areas. We investigated this by analyzing information transfer in a simplified V1-V2 model presented with natural images [95,96]. In the V1-V2 network simulations, we employed isotropic connectivity within V1 and a convergent isotropic Gaussian connectivity between V1 and V2 network models (Fig 7A, top, and Materials and methods). The input to the V1 subnetwork represented parts of a natural image (Fig 7A, bottom). Synchrony was once again uniform across our model V1 during the transient but decayed over distance in the sustained phase (Fig 7B). To quantify the information transfer from V1 to V2, we calculated the average activity in excitatory V1 neurons that connected to a V2 neuron just before that V2 neuron spiked, i.e., a synpatically confined spike-triggered average (_syn_STA; see Materials and methods). Fig 7C and 7F show the resulting V1→V2 _syn_STA maps calculated for V2 spiking in the transient (left) and sustained (right) periods. Remarkably, the V2 RFs visible in the _syn_STA maps were significantly larger in the sustained period compared to the transient period. The V2 RF could also move to a different location (Fig 7C and 7F). The _syn_STA maps represent the effective (functional) RF of V2. Fig 7D and 7G illustrate that the effective RF of a V2 neuron can differ significantly from its anatomically defined feedforward RF, which was isotropically Gaussian in these simulations (i.e., the connection pattern from V1 to V2).

Our simulations show that in the transient phase, the magnitude of _syn_STA strongly matched the V1 spike rates (set mainly by the luminance values of the natural image pixels). This led to a strong bias of effective RFs towards image regions of high input. In contrast, during the sustained period, the produced _syn_STA maps were strongly biased towards regions of high gamma synchronization (see Fig 7D and 7G). Typically, these were regions corresponding to parts of the image where luminosity was very homogenous with the center of the V2 RF. V1 neurons with similar spike rates (leading to low detuning) were more likely to synchronize and were thus more effective in driving V2 neurons. Therefore, during the sustained period, the spatial input homogeneity within a natural image patch is a central component in shaping the effective RFs in V2. Fig 7E–7G illustrates an opposite example: the region of high input strength coincided with high input homogeneity, including the center of the V2 RF, resulting in similar transient and sustained _syn_STA maps.

In summary, the V1 influence on V2 neurons was biased by local stimulus strength during the transient and by spatially homogenous input during the sustained period. Note that in some cases, neurons with a lower intrinsic firing rate had a stronger effect on the downstream population than those with a higher intrinsic firing rate. This occurred for image regions with low, nearly uniform input strength and consequently high neural synchrony (e.g., see the _syn_STA map in Fig 7C extending over the dark region, rather than the high-intensity light region). Thus, in the sustained period, the interplay between stimulus input and local connectivity caused a mode of gamma synchrony that strongly influenced communication to downstream areas.

**Fig 8:**
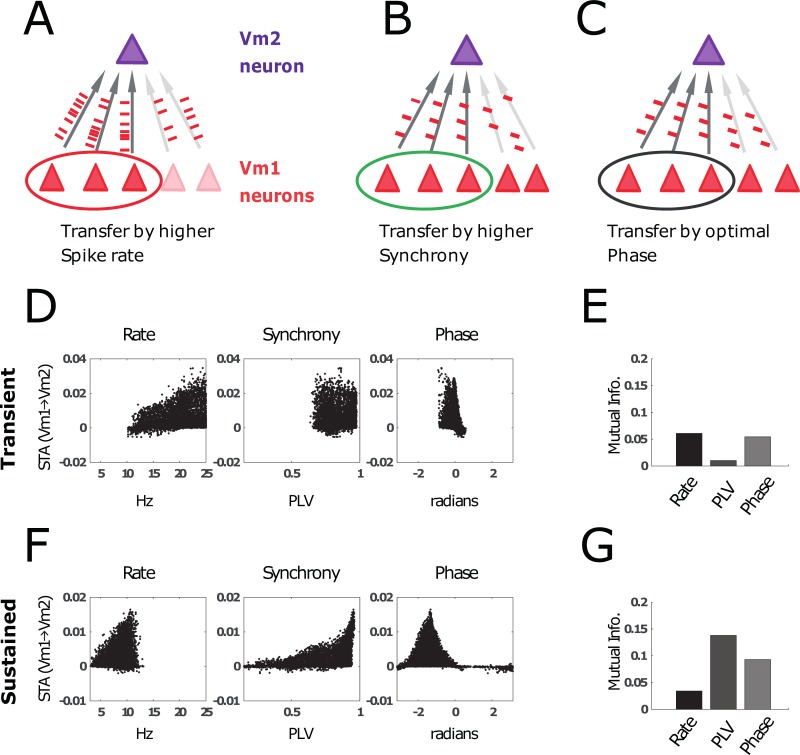
Information transmission (quantified by the STA, see Materials and methods) from V1 neurons to V2 neurons. (A–C) Schematic illustration of factors that could modulate the influence of a particular V1 neuron on a V2 neuron. We compare the information transfer in the transient and sustained period if the activation of V2 is mostly determined by higher spike rates (A), local synchronization (B), or optimal phase differences (C) in V1. (D) Scatter plots from simulations using 100 different natural images. _syn_STA is plotted as a function of (left) spike rate, (middle) synchrony, (right) gamma phase difference. Note: Because the transient is relatively short, “phase” is equivalent to spike latency relative to the saccade onset. (E) We used (normalized) MI to quantify how much of the variance in _syn_STA between V1 and V2 neuron is explained by the V1 neuron’s spike rate, synchrony, and phase. In the transient period, the transfer entropy was best explained by spike rate and phase. (F) Similar to panel D, but for the sustained period instead of the transient. (G). Similar to panel E, but the sustained period was analyzed instead of the transient. Here, the _syn_STA is mainly explained by synchrony and phase. This shows that synchrony-dependent organization in the sustained period has strong effects on feedforward information transmission. MI, mutual information; _syn_STA,.

In the previous sections, we have shown that stimulus information can be encoded in multiple ways. Firstly, stimulus information can be reflected in the spike rate (_syn_STA maps correlate with input during the transient in Fig 7C and 7F). Secondly, stimulus information is visible in the patterns of gamma synchrony (_syn_STA maps correlate with gamma synchrony during the sustained phase in Fig 7C and 7F). Finally, gamma phase of local gamma can be altered by stimulus properties (S5 Fig, S6 Fig). Fig 8A–8C schematically visualizes these 3 coding schemes. In each of the 3 panels, the 3 V1 neurons on the left can communicate better with the V2 neuron. This is either because (A) their firing rate is higher, (B) their firing is more synchronized, or (C) their spikes arrive when the V2 neuron is in a more excitable gamma phase. The STA between the excitatory spikes in V1 and the excitatory spikes in V2 was calculated for the transient (D, E) and sustained (F, G) periods, similar to Fig 7C and Fig 7F. The _syn_STA is plotted as a function of firing rate (left), gamma synchrony (middle), or phase relative to V2 (right). Fig 8D–8G shows the transmission efficacy, estimated by mutual information (MI), of these 3 schemes in the transient and sustained period. This analysis suggests different optimal mechanisms for information transfer in the transient and the sustained periods. In the transient phase, the firing rate is high and predictive of the _syn_STA. Moreover, the phase of firing also predicts the _syn_STA estimate, corresponding to a latency code (Fig 8E, also see Fig 3D). In the sustained period, firing rates are lower and seem therefore less effective for information transmission. By contrast, synchronous firing becomes more relevant as indicated by the high MI between PLV and _syn_STA (Fig 8G). The right panels in Fig 8D and Fig 8F show that the phase distribution is broader and that there is an optimal phase (between −2 and −0.5 radians) in which _syn_STA is maximized. In summary, the transient and sustained modes of activity differ in how neuronal spike output can affect receiving neurons. In the transient period, neurons are most effective when they have a high rate and short latency (synchrony is always high). In the sustained period, neurons are favored when they engage in sustained gamma synchronization and their spikes arrive at an optimal phase in V2.

## Discussion

In this study, we investigated the impact of MSs on neural synchronization and the implication for information transfer using a computational model as well as data from recordings in monkey early visual cortical areas. The network simulations showed oscillatory dynamics locked to MSs, replicating those recorded in monkey V1 and V2 (Fig 1, Figs 4 and 5) [27,53]. Our network simulations suggested a disparity between transient (0–100 ms) and sustained (100 ms onwards, ends at onset of the next MS) postsaccade periods, characterized by different modes of activity. In the transient period, broadband synchrony was high within and between cortical regions. Information transfer was mainly conducted through the order in which neurons fired (latency code) as well as the neurons’ firing rate (Figs 3, 4 and 8). By contrast, in the later, sustained period, changes in gamma synchronization became critical for modulations in information transmission. Late-phase narrow-band gamma, in addition to being stimulus sensitive, was also modulated by local (recurrent) connectivity patterns (Fig 3, Fig 4). Together, these findings suggest that different periods within the MS rhythm are associated with fundamentally different synchronization and coding properties, shedding light on how visual processing is modulated by MSs.

### Two different modes of neural synchrony

Neural synchrony can be divided into evoked transient synchrony and induced narrow-band synchrony [97,98]. Transient broadband synchrony has been widely studied after stimulus onsets [78,99] and after saccades [77,100] as well as after MSs [27,28]. Transient synchrony decays quickly after stimulus or (micro)saccade onset and is gradually replaced by narrow-band rhythmic synchrony. Here, we will focus on gamma-band synchrony that is prevalent in cortical area V1 and V2. In V1 and other visual areas, it has been shown that MSs induce evoked responses similar to stimulus-onset evoked responses [5,20,21,27,53]. As shown in Fig 1, both transient synchrony and induced narrow-band synchrony were nested within a 3- to 4-Hz MS rhythm.

In line with earlier experimental observations in monkey V1 [21,22,27,53,72,101], we observed the quasiperiodic occurrence (every 200–500 ms) of MSs during normal awake visual processing. They continuously modulated visual neuronal processing and prohibited the network dynamics from reaching an equilibrium state. These observations are in line with results from (macro)saccade behavior [6]. MSs and saccades do indeed not only share a similar timescale but also largely share their underlying neural circuitry [5,6,10,11,42].

The (micro)saccade-linked effects on neural activity likely explains why gamma activity, while lengthy enough to be visible as a narrow-band oscillation, has been reported as being transient or “bursty” during single-trial recordings in the visual cortex [102,103]. This has also been shown in the prefrontal cortex [104] and hippocampus [105]. Therefore, nontransient oscillations occur only for a limited time within the intersaccade intervals (approximately 150–400 ms after the saccade onset; see Fig 1B).

The short-lived nature of gamma oscillations and synchronization has important implications for how they might operate and how they should be analyzed [52,53]. For example, studies testing the role of gamma in vision and higher cognition should account for the short-lived nature of gamma and separate it from transient dynamics that likely also have power in the gamma band. As we have shown here in the case of MSs, these transients can occur continuously at a 3- to 4-Hz rhythm in awake animals during visual fixation.

#### Transient synchrony

A feature of transient synchrony occurring after (micro)saccades or stimulus onset is that the triggering event is largely shared between neurons in a cortical area. In part, this is the case because the retinal image shift induced by the eye movement will affect most neurons along the retinogeniculate pathway [33]. This causes a “common reset,” during which a large part of the neural population within the cortical area is either strongly activated or inhibited at the same time [106]. In addition, others have suggested that a corollary discharge associated with the eye movement plays a role in the (micro)saccade-evoked responses observed in visual cortex [15,22,107]. Most likely, both the “retinal refresh” and the “corollary discharge” [8,15,22,24] accounts contribute to the transient synchrony after MSs. Thus, although the “reset” effects seen in the neural activity in visual cortex related to either (micro)saccades or the onset of stimuli may bear many resemblances, their underlying mechanisms may differ at least in part. This is supported by the observation that, even with zero contrast (or in the dark), MS-induced responses can be observed in visual cortex, likely due to corollary discharge [22,23,100,107,108].

It is currently debated whether the transient response evoked by stimulus onset and its accompanying transient synchrony occur due to pure phase reset of ongoing cortical activity or due to an evoked component adding to the ongoing cortical activity [109–112]. The former should result in phase alignment, whereas the latter should result in added spectral power. In our assessment, both views are complementary. Stimulus change or (micro)saccades both lead to an increase in the retinogeniculate input drive and thus to additional energy in V1 networks in line with the latter view. In addition, a strong volley of neural activity arriving in V1 will necessarily interact with the ongoing network dynamics and change the phase of ongoing oscillatory activity.

Transient synchrony does not depend on stimulus parameters as critically as sustained synchrony. A transient stimulus-onset response (the visual evoked potential [VEP]) can be observed reliably across different stimulus conditions and natural images [113]. Nevertheless, stimulus luminance contrast affects (micro)saccade-induced VEP amplitude and latency [114], as does the stimulus-onset VEP [115–117]. Generally, strong transient synchrony occurs when there is an important change in the field of view, as induced by a saccade, and in these cases, a strong and fast feedforward drive would be especially beneficial.

The fact that VEP latency depends on stimulus contrast has led to the idea of a latency code [89,114,115,118,119]. The emergence of the latency code can be understood as follows [120]: assuming that the neurons have similar excitability (membrane voltage) and membrane constants, the time it takes for a neuron with constant excitatory drive to fire an action potential depends on the strength of that excitatory drive. Neurons with stronger excitatory input, e.g., due to higher image contrast in their RF, will spike earlier compared to neurons receiving weaker input, even if the onset of their input is the same. Our modeling and experimental V1 data are in line with this view. The reliable effect on latency of the first spike by sensory variables has been suggested to be essential for rapid initial visual processing [67,68,70,121].

#### Sustained gamma synchrony

The later-occurring gamma-band synchrony is not as short-lived as the transient evoked responses and exists for multiple cycles, so that it can be revealed as narrow-band gamma activity in electrophysiological measures [122]. Narrow-band gamma synchrony depends critically on neural interactions (particularly with local interneurons [75,123]). This is further supported by observations that V1 gamma synchrony is confined by the spread of horizontal connectivity, as shown in Fig 3 [80,124–126]. Likewise, gamma-band synchronization between cortical regions has been shown to rely on mutual interactions to enable phase coordination [29,86,92,127–129]. In addition to local neuronal interactions, gamma synchrony is highly dependent on the level of local neuronal excitation. Therefore, the frequency of gamma-band activity in early visual cortex depends on stimulus parameters and their spatial patterns [80,82,83,125]. This suggests that gamma synchronization may be highly linked to local image characteristics and is limited in both space and time to small populations and MS-dependent time windows. Therefore, the opportunity to measure gamma will be stimulus dependent, which may, in turn, explain why gamma has been observed with natural images in some studies [72] but not in others [130].

### Functional implications

Synchrony and rhythmic spike activity can only be important for brain processes if receiver neurons are sensitive to it [94,131]. Whichever aspect of a stimulus that is encoded in synchronization ultimately must be sent to a receiver network in the form of action potentials. The effect of synchronization on neuronal spiking is supported by several properties of brain networks. Neurons in the brain are heavily interconnected [132,133], and a single synaptic potential will rarely generate a spike in a receiver neuron. Therefore, coincidence of multiple synaptic potentials is required [134]. This fits with observations that precise spike timing can reflect a neural code [120,135–137] and is critical for plasticity [138–140]. Direct experimental evidence for the importance of precise spike timing has been provided for connections between LGN and V1 [141] and between V1 and V2 [142].

Our modeling and empirical results show that the coincidence of spikes plays a role in both transient and sustained neural synchronization. However, we found that during the transient period, crossareal information transfer was modulated mainly by spike latency (phase) and spike rate, whereas during the later sustained period, crossareal information transfer was importantly shaped by level of synchronization (Figs 5–8). This suggests that visual cortex may rely on different mechanisms to optimize information transfer in the 2 saccade-locked modes of neural synchrony. In the next sections, we discuss the functional implications of MS-dependent modulation of neural synchrony and possible coding schemes.

#### Within-area synchrony and feature binding

Previous work has suggested that (narrow-band) gamma rhythmicity is important for stimulus feature grouping [125,126,143,144] or tracing of contours [145]. Gamma synchrony in V1 depends on the horizontal connectivity structure [125,126,144] as well as on particular stimulus properties [80,81,83]. Both properties make gamma rhythms a potentially useful mechanism to locally coordinate visual processing. Our experimental and modeling results show that neurons during the MS-induced transient are highly synchronous over large cortical distances, whereas gamma-band synchrony depends on both local connectivity and stimulus properties. From our findings, we hypothesize that the information that is transmitted during the initial transient phase is mainly feedforward and coarse, whereas subsequently recurrent input (e.g., V1 horizontal connections) becomes more dominant, which may facilitate more detailed contextual operations such as feature binding and contour grouping.

In line with these interpretations, others have found that contextual processing in V1 occurs in the sustained period after the initial transient response related to stimulus onset [146–148]. The same was observed for attention modulation in visual cortical areas [149,150]. This further supports that narrow-band gamma synchronization following MSs is associated with contextualization of incoming sensory input. In addition, we hypothesize that transient responses represent a tool for disrupting “old interpretations” (in form of temporal and spike rate patterns) and replacing them by new input and new interpretations. In other words, (micro)saccades may be important for enabling perceptual and cognitive flexibility.

#### Crossareal communication and RFs

Our results suggest that the MS-dependent modulation of neural synchrony has significance for crossarea communication and the shaping of the effective RF [8,151–156]. Our experimental V1-V2 analysis showed that only the narrow-band gamma synchronization within the later part of the MS interval was selective for stimulus properties and the RF distance between V1 and V2 neurons. Our modeling showed (Fig 7) that, in the sustained period, the RF of our V2 model (defined here as the neurons in V1 that influence V2) was different from the RF in the transient period. The V2 RF during the sustained period depended on stimulus properties as well as local connectivity and gamma rhythmicity [142]. These findings are in agreement with experimental evidence for stimulus-dependent RFs [157,158]. This calls for a dynamic view of RFs in which the sensitivity to subregions of the RF is shaped by synchrony of upstream neurons. This leads to potentially significant RF differences between periods of transient and sustained synchrony.

The importance of local synchrony in the sending neuronal population for information processing in the receiving population (see Fig 7 and Fig 8) is in line with existing literature. That MSs have been linked to both the shaping of crossareal synchrony [27,53,94,159] and attention [8,40,41,160] is consistent with the work presented here. Future work on the effects of MSs on crossarea synchrony may help in understanding how crossareal synchronization is modulated during attention [69,161].

#### CTC and CFC

Our work is compatible with the “communication through coherence” (CTC) framework [93,94,159,162–164], stating that coherence (phase coordination) is a critical dynamical mechanism for the control of crossareal information flow. Our work also emphasis the role of cross-frequency coupling (CFC [57,58,69,165]) in the regulation of synchrony-modulated information transfer. Our work showed that crossareal synchrony and its implications for information transfer differ importantly depending on the timing within the MS interval. However, in many studies using the common visual fixation paradigm, these different synchronization periods are averaged together. To distinguish the 2 different periods, an accurate marker of this transition is very important. A critical advantage of MS timing is that they can be relatively precisely estimated without any assumption of the underlying rhythmicity. We therefore encourage the use of both MS timing and LFP lower-frequency rhythms to understand CTC and CFC in visual cortex.

Furthermore, crossarea CTC is mainly investigated in the context of attention, such that selective attention modulates synchrony between cortical location as a function of attentional location [69,151,166]. Here, we show that CTC occurs (automatically) during stimulus processing, in line with the “binding-by-synchrony” hypothesis, which, however, focused mainly on stimulus feature-dependent within-area synchrony. Our experimental and modeling work suggests and encourages integrating within-area and between-area synchrony within a common theoretical framework. Moreover, selective synchronization is a basic property of neural network interactions that likely plays a role in general stimulus processing as well as attention or other cognition processes.

#### MS-modulated synchrony and visual input transformations

Recent work has shown that MSs, in tandem with drifts, have important implications for transforming visual spatial information into temporal modulations [35,36,50,51,101]. MSs induce transient modulations that relate to lower- and high-frequency content of the visual image, whereas drifts lead to temporal modulations related mainly to higher-frequency (more local) content. Our work is, in principle, compatible with and complementary to these findings. MS-induced transient synchrony is widespread over the cortical space, and we hypothesize that this might support processing of more global or spatial low-frequency features of the image. Alpha-beta rhythms, which are synchronized over a wider cortical space and reset by (micro)saccades [25,167,168], might play a particular role here. In the later part of the MS interval, synchrony becomes more local and dependent on local stimulus features. The gamma band might be critical for processing of local and high-frequency content of the image, particularly at the level of surround RF interactions [154,155]. The relation to drifts is, however, unclear. Drifts has been shown to modulate firing rate of V1 neurons [26] and to prevent fading in conjunction with MSs [37]. Changes in local population firing rate can shift the preferred frequency of gamma rhythms, and the specific frequency is critical for the regulation of gamma synchrony [29,90,127,169]. We therefore predict that drifts might systematically shape the cortical gamma synchronization patterns (phase-locking and phase-relation), not via resetting but through modulations of the precise frequency. The relation of MS, drifts, synchrony, and visual coding requires further investigation.

### Model caveats

It is important to note some limitations of our model. First, parameters such as input strength, neural noise, and connectivity strength were not explicitly constrained by experimental data. This means that predictions are of a qualitative nature. Our model of visual cortex lacks synaptic interactions with long timescales, such as gamma-aminobutyric acid B (GABA-B) and N-methyl-D_aspartate (NMDA) synapses or synaptic plasticity. How exactly these slower channel dynamics interact with the MS-dependent activity studied here needs further investigation.

It is not well understood whether the observed changes in neural activity locked to a saccade are due to the rapid visual input change on the retina, due to a corollary discharge (efference copy [5,8,15,22,23,170]), or due to a mixture of both. Our model does not attempt to disentangle these possibilities.

Our model did not include crossarea feedback connections or explicit top-down signals. Experiments in monkey V1 in the context of figure-ground segregation [147] suggest that feedback signals in response to a stimulus from other cortical or subcortical areas arrive >100 ms after stimulus onset. The crossarea feedback signals related to the new image on the retina after a saccadic eye movement will likely follow a similar timescale. This would mean that crossarea feedback signals would add to the influence of the horizontal connections during the sustained period and can have an additional influence on the RF. Finally, the predictions from our model are not specific with regard to cortical layers. Others have suggested that feedforward, feedback, and horizontal connections mainly project to different layers [147,171,172]. This could mean that the transient and sustained activity modes are separated in space (cortical depth) as well as in time.

Finally, the input to the network was simplified to make the gamma synchronization effects more clear in the simulated activity. To generate the coherence plots in Fig 2, Fig 3 and Fig 7 as well as the STA maps, the simulated network was presented with a repetition of the same stimulus (besides time-varying noise; see Materials and methods). This means that the input directly before each MS was very similar across repetitions, potentially affecting the postsaccade dynamics of the network. Because in reality a (micro)saccadic eye movement will always lead to a change in retinal input, we ran another simulation similar to Fig 2. This time we presented 3 different input patterns in a random order (S3 Fig), thereby accomplishing a more naturalistic variation of pre- and postsaccadic inputs. This did not affect the model results, indicating that the transient gamma was effective in resetting the network dynamics prior to the beginning of sustained gamma. A more systematic empirical investigation of the effects of presaccadic input may elucidate to what extent a (micro)saccade is able to reset the state of the neuronal dynamics.

Other effects such as drift of the retinal image between saccades were not explicitly modelled. These were only implicitly included through the Gaussian noise in the input current.

### MSs and saccades

The question of whether our findings are applicable to saccades—which during natural exploration are more dominant [4,6,11,42,173]—arises. Saccades and MSs share largely the same underlying neural circuitry [6,8,10–16] and exhibit similar temporal (spectral) properties [15,28,67,72,174]. This suggests that our findings obtained with MS will be generalizable to saccade-related cortical dynamics. However, future studies investigating MS and saccades together need to test whether differences in modulation of synchrony exist. We focused on MS as they occur during our visual fixation paradigm. This is particularly relevant because visual fixation is a very common and central paradigm used to study visual processing, attention and other cognitive processes, and pathological conditions in monkeys, as well as in humans.

### Endogenously generated oscillations and active sensory sampling

The rhythmicity in the occurrence of (micro)saccades is endogenously generated in the brain and therefore has to be distinguished from external sources of rhythmicity occurring in the natural environment. The neural circuits underlying the rhythmic pattern of eye movements (e.g., frontal-parietal cortex, superior colliculi, brain stem) should therefore contain oscillatory activity patterns at the same frequencies. It has been found that the LFP delta-theta 3- to 4-Hz oscillations in the visual cortical areas V1, V2, and V4 [27,53] are closely related to the MS rhythm as well as the rhythm of larger saccades [28,100]. However, it is unclear whether the neural generator of the LFP delta-theta rhythms and that of the (micro)saccade rhythm are identical, overlapping, or distinct from each other. The observation that LFP rhythms and MSs are strongly related to each other, as well as the widespread synchronization of theta rhythms across cortical and subcortical areas, suggests that different theta rhythmic processes are well coordinated in the brain and might at least partially share their underlying generation mechanism.

Related to these open questions is the issue of whether neural activity patterns associated with (micro)saccades are due to retinal image change, corollary discharge, or linked to delta-theta rhythms, which are synchronized to the MS rhythm. Future studies need to tackle these important questions.

### Outside visual cortex

Our model is based on processing in early visual cortex. One may ask whether a similar mechanism of rhythmic switching between a transient and sustained mode of neural activity may exist in other cortical areas. It is possible that other early sensory areas may exhibit similar dynamics, especially in the context of Active Sensing [3]. One example is sniffing behavior when considering olfaction. Sniffing is a rhythmic (3–7 Hz) activity responsible for active sampling of olfactory information and is reflected in neural responses [2,73]. Similarly, whisker movement in rodents has been shown to actively sample somatosensory information in a rhythmic manner [1,74]. Like the MSs in our model, sampling actions such as sniffing and whisking will cause transient activity in the relevant sensory cortex [7]. In the interval between the sampling actions, there would be time for the neural activity to show the sustained dynamics, similar to the sustained activity during the intersaccade interval in early visual cortex.

A trigger for rhythmic switching between sustained and transient modes could also be provided by neural network oscillations in the delta-theta range (1–10 Hz [175]) without the need of sensory organ movement. The phase of these slow rhythms has been shown to modulate neural activity [105,176] and is possibly related to an attentional rhythm [62,63,65,177]. In visual cortical areas, MS rhythm and LFP oscillations work in tandem [27,53]; however, the LFP oscillations might still enable switching of sustained and transient modes even in the absence of MSs.

Taken together, our work outlines a fundamental rhythmic switching mechanism between feedforward and local processing during active sensory processing.

## Material and methods

### Ethics statement

Two adult male rhesus monkeys were used in this study. All the procedures were in accordance with the European council directive 2010/63/EU, the Dutch “experiments on animal acts” (1997) and were approved by the Radboud University ethical committee on experiments with animals (Dier Experimenten Commissie [DEC]).

### Neuron model

We used a 2D integrate-and-fire neuronal model introduced by Izhikevich [178], extended with exponentially decaying synapses. The dynamics of neuron *i* are given by Eqs 1 through 4.

$${\dot{V}}_{i}=0.04{V_{i}}^{2}+5V_{i}+140-u_{i}+I_{i}(t)$$ (1)

$${\dot{u}}_{i}=a(bV_{i}-u)$$ (2)

$${\dot{s}}_{i}={-s}_{i}/\tau_{i}$$ (3)

$$\mathrm{if}\;V_{i}\geq 30\;\mathrm{mV},\;\mathrm{then}\left\{ \begin{array}{c} V_{i}\leftarrow c\\ u_{i}\leftarrow u_{i}+d\\ s_{i}\leftarrow 1 \end{array}\right.$$ (4)

*V*_*i*_ represents the neuron membrane potential, whereas *u*_*i*_ represents a membrane recovery variable. The membrane potential is reset to the value of *c* (and thus an action potential is said to be generated) when it reaches a threshold value (30 mV). Parameters *a*, *b*, *c*, and *d* are taken from previously published work [178] for the 2 different types of neurons used: RSs and FSs (see Table 1). The dynamics of the presynaptic gate are denoted by *s*_*i*_. The total input to neuron *i* (*I*_*i*_) consists of the sum of the synaptic inputs, together with any imposed input currents and their noise.

$$I_{i}(t)=\sum_{j}s_{j}g_{i,j}(V_{\mathrm{rev},j}-V_{i})+I_{i}^{imp}(t)+\eta (t)$$ (5)

**Table 1:** Parameters for the RSs and FSs.

	RS	FS
*a*	0.02	0.1
*b*	0.2	0.2
*c*	−65	−65
*d*	8	2
τ	10 ms	5 ms
*V*_rev_	50 mV	−90 mV

Abbreviations: FS, fast-spiking neuron; RS, regular-spiking neuron.

Here, *V*_rev_ is the reversal potential of the synaptic connection, and the conductance-like factor *g*_*i*,*j*_ is the synaptic connection strength between neuron *j* and *i*. Both the time constants τ_*i*_ and the reversal potentials *V*_rev,i_ depend on the type of synapse (AMPA or GABA) and therefore depend on the type of the presynaptic neuron (see Table 1).

The equations describing the neuronal dynamics (1–4) were numerically integrated using the Runge-Kutta method [179] with a time-step of 0.5 ms. In Eq 5, the noise *η* on the imposed input current was sampled in each simulated time-step from a normal distribution. The standard deviation of this normal distribution was equal to the square root of the strength of the imposed input current divided by an SNR factor (see Eq 6). The SNR factor was simulation specific).

$$\left(\eta (t)∼N(0,\sqrt{\frac{I_{i}^{imp}(t)}{SNR}}\right)$$ (6)

#### Imposed input currents

The network of neurons received an imposed input current mimicking synaptic input from the thalamic LGN. The input current consisted of 2 factors:

$$I_{i}^{imp}(t)=J_{i}\cdot \varsigma (t)$$ (7)

In the above equation, *J*_i_ is neuron dependent, and it corresponds to the current representing the stimulus input pattern in a retinotopic sense. The time-dependent term *ς*(*t*) is the MS modulation function, which was generated by convolving a string of delta functions separated by intersaccadic interval *T* = 400 ms with an MS modulation kernel:

$$\varsigma (t)={Ш}_{T}(t)*K(t)$$ (8)

Here, Ш_*T*_ denotes the Dirac comb function with period *T*:

$${Ш}_{T}(t)=\sum_{k=-\infty }^{\infty }\delta (t-kT)$$ (9)

The function *δ*(x) is the Dirac delta function (i.e., *δ*(*x*) = 0 for *x* ≠ 0 but ∫ *δ*(x)*dx* = 1 for interation intervals containing *x* = 0). The index *k* takes all integer values. *K(t)* is the MS modulation kernel:

$$K(t\prime )=\left\{ \begin{array}{c} 1+\frac{M_{P}}{Z_{P}}*(\exp\left(-\frac{t^{\prime }}{\tau_{1,P}}\right)-\exp\left(-\frac{t^{\prime }}{\tau_{2,P}}\right))\;\mathrm{if}\;t\geq 0\\ 1-\frac{M_{N}}{Z_{N}}*(\exp\left(\frac{t^{\prime }}{\tau_{1,N}}\right)-\exp\left(\frac{t^{\prime }}{\tau_{2,N}}\right))\;\mathrm{if}\;t<0 \end{array}\right.$$ (10)

The decay constants *τ*_1,*P*_ and *τ*_2,*P*_ control the length of the transient, and *τ*_1,*N*_ and *τ*_2,*N*_ control the length of the presaccadic inhibition (with *τ*_1,*x*_ > *τ*_2,*x*_ for *x = P*,*N*). The factors *M*_*P*_ and *M*_*N*_ control the strength of the transient and the presaccadic inhibition, respectively. The normalization factor *Z*_*x*_ with *x* = *P*, *N* is:

$$Z_{x}={\left(\frac{\tau_{2,x}}{\tau_{1,x}}\right)}^{\frac{\tau_{1,x}}{\tau_{2,x}-\tau_{1,x}}}-{\left(\frac{\tau_{2,x}}{\tau_{1,x}}\right)}^{\frac{\tau_{2,x}}{\tau_{2,x}-\tau_{1,x}}}$$ (11)

The MS modulation parameters are given in Table 2.

**Table 2:** Parameter values for the MS modulation kernel (see Eq 10).

*τ*_1,*P*_	*τ*_2,*P*_	*τ*_1,*N*_	*τ*_2,*N*_	*M*_*P*_	*M*_*N*_
100 ms	40 ms	15 ms	10 ms	0.5	0.2

Abbreviation: MS, microsaccade.

Convolving the kernel with these parameter values leads to the MS modulation function *ς*(*t*) illustrated in Fig 1A and used in Fig 2. Note that others have shown that MS modulation varies spatially across the visual cortex depending on the MS’s direction [24]. In our simplified model for MS modulation, we have chosen to omit these effects.

For Fig 3A, the mean input was an oriented bar-shaped input pattern to illustrate its influence on the spatial distribution of gamma synchronization. The shape was constructed from a 2D thresholded sinusoidal function with added noise:

$$J(x,y)=\Theta \left[\cos(\left(1+a)\pi \;(\frac{x}{L}-\frac{1}{2}\right))\cos\left(\left(1+b)\pi \;(\frac{y}{L}-\frac{1}{2}\right)\right)-0.5\right]$$ (12)

$$*0.5*(\cos\left(4\pi \frac{ax+by}{L}-\frac{1}{2})+1\right)$$

In the above equation, Θ[*x*] is the Heaviside step function (i.e., Θ(*x* ≥ 0) = 1 and Θ(*x* < 0) = 0). *L* is the size of the neuronal grid (in this case, *L* = 40). The factors *a* and *b* determine whether the stimulus is oriented horizontally or vertically. For the stimulus in the top row of Fig 3A, *a* = 1 and b = 0. For the bottom row, *a* = 0 and b = 1.

In Figs 7 and 8, we used patches of natural stimuli from the Berkeley segmentation dataset (BSDS500 [95,96]). The luminance of every pixel of a 40 × 40 patch determined the mean input strength of the corresponding neuron (*J*_*i*_ in Eq 7). The luminance values were first normalized to be between 0 and 1. After this, the input to the E neurons was multiplied by a factor 7, whereas the input to the I neurons was scaled by 3.5. Note that for illustration purposes, we chose luminance as the main feature that determined input drive to our visual cortex model. However, the conclusions in the current work are not affected by whether luminance or local contrast is chosen.

#### Neural connectivity

The synaptic connection probability depended on their Euclidean distance *D*. Note that we employed periodic boundary conditions when calculating D. This means that a neuron on the left edge of the grid is a neighbor of both the neuron directly to the right as well as the neuron on the right edge of the grid. The same holds for neurons along the top and bottom edges. All connection patterns were generated using Gaussian distributions.

$$P(D)=\frac{1}{Z}exp(-\frac{D^{2}}{2\sigma_{S}^{2}})$$ (13)

Here, *σ*_*S*_ determines the reach of the neuronal connections; this value changed between simulations (see below). *Z* is a normalization factor. The probability of a neuron to connect to itself is set to 0.

Every neuron received a fixed total number of inputs, *N*_*S*_, all of which had the same strength *g*_*s*_. The parameters *N*_*S*_, *g*_*S*_, and *σ*_*S*_ (in Eq 13) were specific to sender–receiver pair cell type (e.g., *N*_*S*_ was different for *S = "*E to I” connections compared to *S = "*E to E” connections; the same holds for *g*_*S*_).

The Gaussian connection probability distribution (Eq 13) was then sampled *N*_*S*_ times to generate the synaptic connections. During this sampling process, a neuron pair could be sampled more than once, linearly increasing the connection strength between that neuron sender–receiver pair (i.e., the total connection strength was then equal to a multiple of *g*_*S*_).

In general, excitatory connections had a longer reach than inhibitory connections. For all network simulations with the exception of Fig 3B, the reach of the excitatory connections (*σ*_*S*_ in Eq 13) was set to 20, whereas inhibitory connections were more local, and *σ*_*S*_ was set to 1.

In the case of the anisotropic network in Fig 3B, connectivity within V1 was the same as in the network used for Fig 2, with the exception of the EE connections. In the case of Fig 3B, only EE connections emerging from the neuron in the center of the network were kept and the strength scaled by factor 3. The EE connection pattern was then spatially restricted by changing the connection-generating distribution (Eq 13) to an oriented bar-like shape, similar to the input pattern in Fig 3A.

### LFP

An LFP signal was approximated by the summation of membrane potentials of nearby excitatory cells. The contribution of a neuron to the LFP signal recorded at a virtual electrode decreased with its Euclidean distance *D* to that virtual electrode according to a Gaussian kernel:

$$K_{LFP}(D)=exp(-\frac{D^{2}}{2\sigma_{LFP}^{2}})$$ (14)

Here, *σ*_*LFP*_ determines the spatial spread of the LFP and was set to 1 neuron distance on the square grid.

#### LFP electrode grid

In the simulations, a 40 × 40 square grid of excitatory neurons was covered by a 10 × 10 grid of virtual electrodes of the same size (i.e., with a distance between electrodes equal to 4 times the distance between excitatory cells).

### _syn_STA

To estimate the effective (functional) connectivity from V1 neurons to V2 neurons, we used an _syn_STA approach. We used the V2 spike as a trigger to define time windows over which to average preceding V1 spike activity. In this way, we computed the V1 spike probability occurring just before a V2 spike. For a given V2 neuron, we computed the above-defined _syn_STA only for those V1 neurons that were presynaptic to the V2 neuron.

$$STA_{i,k}(\tau )=\frac{1}{N_{j}}\sum_{t=1}^{T}\sum_{i\in K}S_{i,j}\nu_{j}(t-\tau )\nu_{i}(t)$$ (15)

Here, *ν*_*i*_(*t*) is the number of spikes produced by V2 neuron *i* at the trigger time *t*, and *ν*_*j*_(*t*−*τ*) is the number of spikes produced by V1 neuron *j* at time *t* − *τ*. *S*_*i*,*j*_ is a binary matrix indicating whether V1 neuron *j* and V2 neuron *i* are synaptically connected. This is summed for all V1 neurons *i* ∈ *K*, where *K* contains all neurons picked up by virtual electrode k. This measure is normalized by the total number of spikes produced by V2 neuron j (*N*_i_) in the trial of length *T*. To get one _syn_*STA* value, we averaged *STA*(*τ*) values from *τ* = −8 ms to *τ* = −2 ms. Finally, we subtracted a shuffled _syn_STA to correct for spurious variation due to spike rate differences. For shuffling, we applied the same analysis as above but chose a random set of time points as triggers.

### MI

We used an MI metric to estimate the mutual dependence between the _syn_STA and properties of the sending neurons such as firing rate, firing phase, and phase locking with its neighbors. The MI of 2 signals (*X* and *Y*) was calculated by binning the signals and constructing discrete probability density histograms (*P*) and calculating the Shannon entropy (*H*):

$$H(X)=-\sum_{x\in X}P(x)\;\log_{2}\left(P(x)\right)$$ (16)

Here, *x* runs over all instants (bins) of the signal *X*. The MI *I*(*X*, *Y*) is defined as

I(X,Y)=H(X)−H(X|Y) (17)

where *H*(*X*|*Y*) is the conditional entropy:

$$H(X|Y)=\sum_{x\in X,y\in Y}P(x,y)\;\log_{2}\;\left[\frac{P(x)}{P(x,y)}\right]$$ (18)

### Spectral analysis

#### TFR

Time-resolved spectral information of the simulated time-series was obtained from TFRs. These were calculated by estimating spectral power of the simulated LFP signals. Short-time Fourier transforms were obtained using a 150-ms sliding Hann window. Zero-padding was used to interpolate the frequency spectra to achieve the frequency resolution presented in the figures.

#### PLV and mean phase

For quantifying consistent synchrony across sites, we employed the PLV [84]. The PLV measures the consistency of the phase difference between 2 LFP signals across saccades using Eq 19:

$${\mathrm{PLV}}_{x,y}=|\sum_{n=1}^{N}\exp\left(i(\phi_{n,y}-\phi_{n,x})\right)|$$ (19)

*N* is the total number of saccades $and\;i=\sqrt{-1}$. The phase of LFP signal *x* in saccade *n* is denoted by *ϕ*_*n*,*x*_. PLVs take values between 0 (no phase locking) and 1 (perfect phase synchrony between sites across saccades). Note that the phase signals *ϕ*_*n*,*x*_ can be a function of time to result in time-resolved PLVs.

The mean phase difference ($\bar{\theta }$) between the sites was computed by taking the argument rather than the modulus:

$${\bar{\theta }}_{i,j}=\arg\;\left(\sum_{n=1}^{N}\exp\left(i(\phi_{n,j}-\phi_{n,i})\right)\right)$$ (20)

### Short summary of the monkey data

A detailed description of the MS-linked spectral gamma dynamics recorded in monkey cortical areas V1 and V2 during visual stimulation can be found in [29,53], where details on ethical approval, surgical preparation, recording parameters, and other information can be found. Here, we use the recorded V1 data that are described in detail in [29].

For Fig 1B, we used LFP data recorded in V1 of one representative session of monkey M1. The data were recorded from a single 16-channel U-probe (Plexon) while gratings (2 cycles/degree, 5 degrees in diameter) were presented that were centered over the recorded RF.

For Figetions with expception of Figs 3B, 4, 5 and 6, we used data from the same animals [29,53]. In contrast to the data shown in Fig 1B, here the data were acquired using 3 simultaneously inserted U-probes consisting of 16 contacts (150-μm intercontact spacing). The 3 probes were arranged linearly, each spaced about 2 to 3 mm apart. Thus, in each session we recorded 2 “near” pairs in which the electrodes were 2 to 3 mm apart. The corresponding local RFs recorded at each probe were approximately 1 degree apart. Secondly, there was one “far” pair that consisted of the 2 electrodes that were 4 to 6 mm apart. This lead to corresponding local RFs that were approximately 2 degrees apart. The task of the monkey was to fixate on a dot on the screen while a full-screen square wave grating (2 cycles per degree) was shown for 2 seconds. The stimulus had spatially varying luminance contrast, such that different RFs received different contrasts. We had different conditions to parametrically manipulate the contrast difference between RFs [29].

For Figs 5 and 6, we also used contacts that were situated in V2, which frequently occurred because the laminar probes were long enough to get part of the V2 lying beneath V1. The method to assign contacts to V1 or V2 has been described in detail in [29]. In short, the transition from V1 to V2 can be easily observed through marked shifts in the RF position and size.

In both experiments (Fig 1B and Figs 4–6), fixation behavior was monitored using a low-resolution eye tracker directed at one eye (Arrington; 60 Hz). The eye tracker was optimized to control saccade behavior and was not sufficient to provide robust and accurate enough MS time estimation. Therefore, in addition, we measured MSs in the other eye by means of an eye-tracking system with high spatial and temporal resolution (Thomas Recording; 240 Hz). For a complete description of the experiments, see [29].

## Supporting information

S1 Fig Distance-dependent effects on synchrony persist when sustained period is lengthened. The input to the neurons in the simulation used to generate was altered by lengthening the MS-modulation kernel. (A) The modified MS-modulation kernel with lengthened interval between MSs. (B) Stimulus input pattern shown to the network. (C–D) The same analysis as displayed in Fig 2C and 2D. (E) A scatter plot showing the PLVs for transient and sustained gamma rhythms (see encircled crosses in C, D, and F) as a function of distance between electrodes. (F) The same analysis as shown in Fig 2E. All the effects demonstrated for the sustained period last for throughout the full intersaccade interval. MS, microsaccade; PLV, phase-locking value.
(PDF)

S2 Fig Experimental analysis of V1 MS-triggered TFR power for different MS intervals. Panels are population averages from 2 monkeys (12 sessions, 36 laminar probes). (A) Averaging only for MS intervals that were between 160 ms and 240 ms. Black line represents averaged eye speed. (B) The same as in panel A, but for MS intervals that were longer than 330 ms. MS, microsaccade; TFR, time-frequency representation.
(PDF)

S3 Fig Gamma-band effects during the sustained period stay intact when varying stimuli across saccades. (A) During each interval between MSs, the network was presented with one of 3 possible stimuli. The stimulus patterns were randomly selected, but no 2 successive stimuli were the same. (C–F) As in S1 Fig. MS, microsaccade.
(PDF)

S4 Fig The 2 different ways of achieving synchrony explained schematically. (A) Synchrony is achieved through (periodic) resetting by an outside source. (B) Synchrony arises through mutual interactions. (C) When synchrony is determined by an outside resetting pulse, connection strength and detuning no longer influence synchrony, therefore the Arnold Tongue is not visible in this case. (D) When synchrony is caused by mutual interactions, the connection strength has to be high enough to overcome any differences in intrinsic frequency (detuning). This results in a triangular region of synchrony also known as “the Arnold Tongue,” shown here.
(PDF)

S5 Fig Arnold tongue characterization of simulated neural activity using a model network similar to that used in from Fig 3. The simulation used for this analysis contained a network with isotropically connected neurons (as in Fig 3 and panel A) with locally varying input strength. (A) In the transient period, coherence (quantified by the PLV; see Materials and methods) was high for any combination of connectivity strength and input difference (left), whereas in the sustained period, PLV was dependent on both input difference and connectivity (right). The synchronization region had a triangular shape known as the Arnold tongue. (B) The same as panel A, but for mean phase difference. PLV, phase-locking value.
(PDF)

S6 Fig Arnold tongue characterization of LFP recorded in monkey V1. Based on the same dataset as the one used for Figs 4–6. Because connection strength and local input drive cannot be measured directly, interaction strength and detuning were used on the 2 axes (see above). LFP, local field potential.
(PDF)

S7 Fig Setup of a V1-V2 model network with anisotropic connection patterns. (A) Left: schematic representation of the network with its 2 subnetworks. Neurons in the first subnetwork (V1; 40 × 40 RSs, 20 × 20 FSs) receive direct input and project to the second subnetwork (V2; 100 RSs, 25 FSs) and to themselves. Neurons in V2 only project locally, i.e., there is no feedback to V1. The local connections in V1 were sampled from anisotropic Gaussians, illustrated by the red oval (see panel B). The feedforward connections (from V1 to V2) and local connections in V2 were sampled from a uniform distribution. Right: an example of the direct input to V1. V1 received one of 16 differently oriented gratings. The orientation (**θ**) of the grating is illustrated by the white overlay. (B) The connections from the center excitatory neuron in V1. Left: connections to other excitatory neurons within V1. Right: connections to inhibitory neurons within V1. (C, D) TFR of the mean LFP power, locked to saccade onset. Average across the electrodes in V1 (C) or for the single electrode in V2 (D). The vertical lines indicate the borders of the transient (0–70 ms, dashed) and the sustained period (200–350 ms, solid white) that were used for analysis in E–G. (E) The mean firing rates for the 2 response periods in V1 and V2. Error bars denote standard deviation across the 50 MSs. (F) The modulation of spike rate (normalized by the orientation average) as a function of stimulus orientation during the transient and sustained periods in V1 and V2. Error bars denote standard error of the mean across the 50 MSs. (G) Orientation sensitivity in panel E quantified by calculating the OSI (see S1 Text). FS, fast-spiking neuron; LFP, local field potential; MS, microsaccade; OSI, orientation selectivity index; RS, regular-spiking neuron; TFR, time-frequency representation.
(PDF)

S1 Text Supplementary information. (1) The effect of increasing MS interval time on the network model and on V1 LFP recording sites. (2) Simulations showing that MS-locked effects persist if stimulus changes after each MS. (3) Proposal of a general theoretical framework to understand MS-locked synchronization changes. (4) Discussion of the theory of weakly coupled theory and the Arnold tongue and demonstration that the Arnold tongue can only be reconstructed in the sustained part of the MS interval in the network model and between V1 recording sites. (5) Additional simulations to illustrate functional implications MS-locked synchronization changes. We show that stimulus orientation sensitivity of a network model differs between the 2 phases within the MS interval. (6) Supplementary methods. LFP, local field potential; MS, microsaccade.
(DOCX)

## Abbreviations

2D: two-dimensional
AMPA: α-amino-3-hydroxy-5-methyl-4-isoxazolepropionic acid
CFC: cross-frequency coupling
CSD: current source density
CTC: communication through coherence
DEC: Dier Experimenten Commissie
FS: fast-spiking neuron
GABA: gamma-aminobutyric acid
LFP: local field potential
LGN: lateral geniculate nucleus
MI: mutual information
MS: microsaccade
NMDA: N-methyl-D_aspartate
PING: Pyramidal-InterNeuron Gamma
PLV: phase-locking value
RF: receptive field
RS: regular-spiking neuron
SNR: signal-to-noise ratio
_syn_STA: synaptically confined spike-triggered average
TFR: time-frequency representation
VEP: visual evoked potential
