# Supplementary figures and images for: Microsaccade-rhythmic modulation of neural synchronization and coding within and across cortical areas V1 and V2
Source: PLoS Biol. 2018 May 31;16(5):e2004132. doi: 10.1371/journal.pbio.2004132 (PMC5997357; doi:10.1371/journal.pbio.2004132)

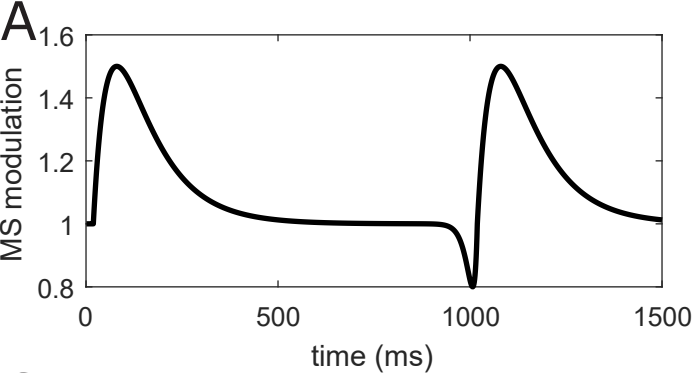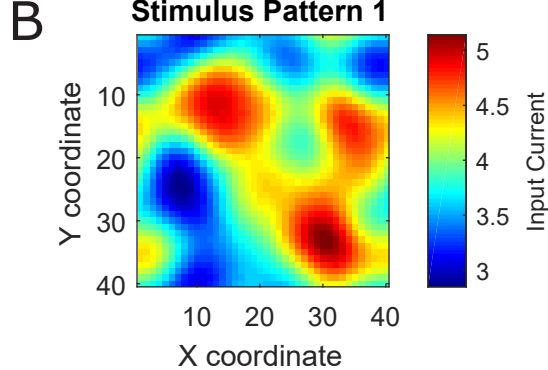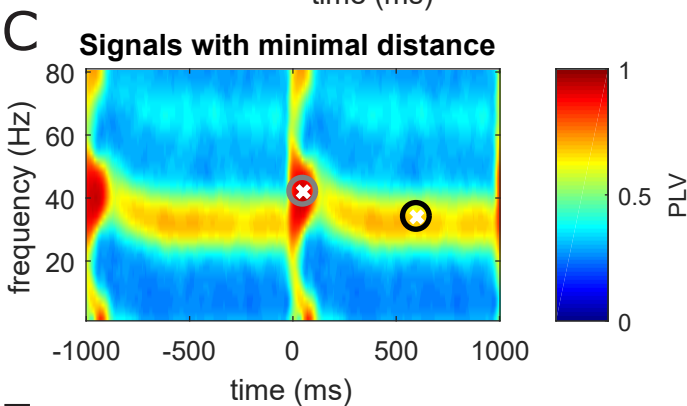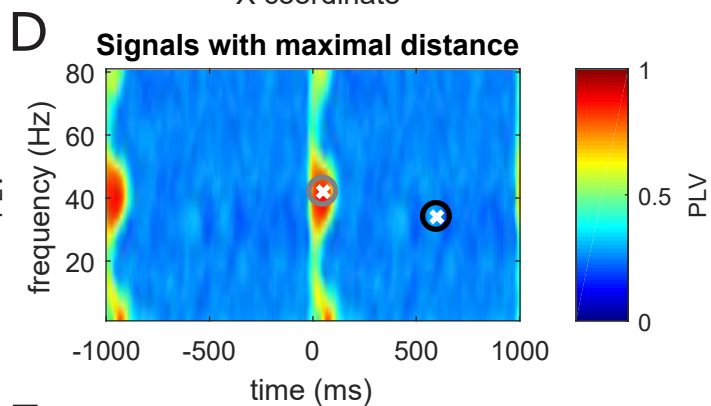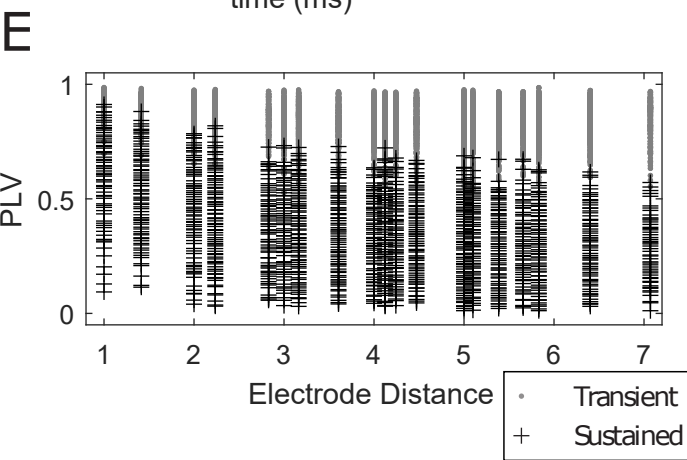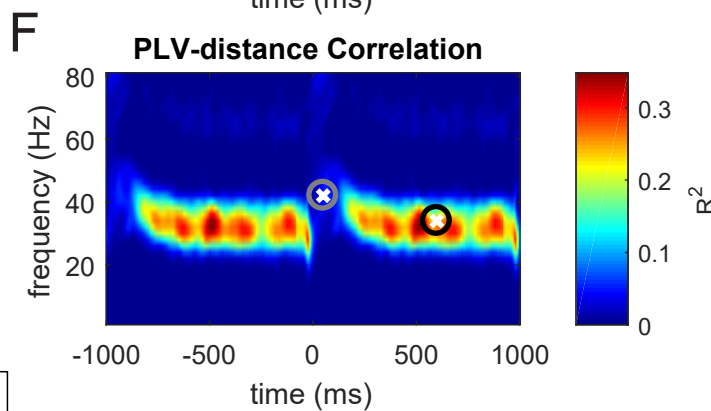

Supplement: S1 Fig — The input to the neurons in the simulation used to generate was altered by lengthening the MS-modulation kernel. (A) The modified MS-modulation kernel with lengthened interval between MSs. (B) Stimulus input pattern shown to the network. (C–D) The same analysis as displayed in Fig 2C and 2D. (E) A scatter plot showing the PLVs for transient and sustained gamma rhythms (see encircled crosses in C, D, and F) as a function of distance between electrodes. (F) The same analysis as shown in Fig 2E. All the effects demonstrated for the sustained period last for throughout the full intersaccade interval. MS, microsaccade; PLV, phase-locking value. (PDF) [file pbio.2004132.s001.pdf]

**A****MS interval: >160ms & <240ms**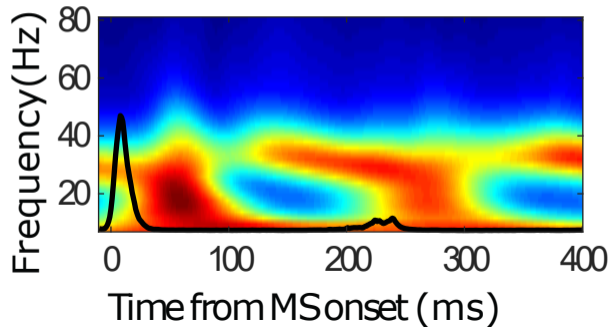**B****MS interval: >330ms**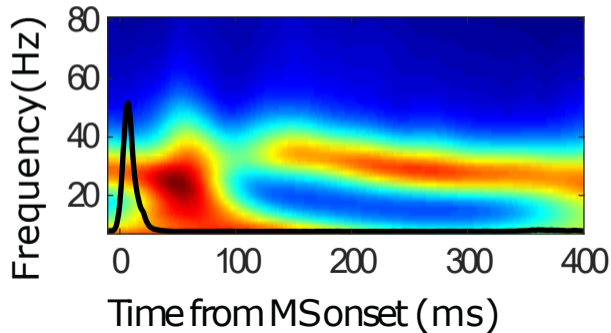

Supplement: S2 Fig — Panels are population averages from 2 monkeys (12 sessions, 36 laminar probes). (A) Averaging only for MS intervals that were between 160 ms and 240 ms. Black line represents averaged eye speed. (B) The same as in panel A, but for MS intervals that were longer than 330 ms. MS, microsaccade; TFR, time-frequency representation. (PDF) [file pbio.2004132.s002.pdf]

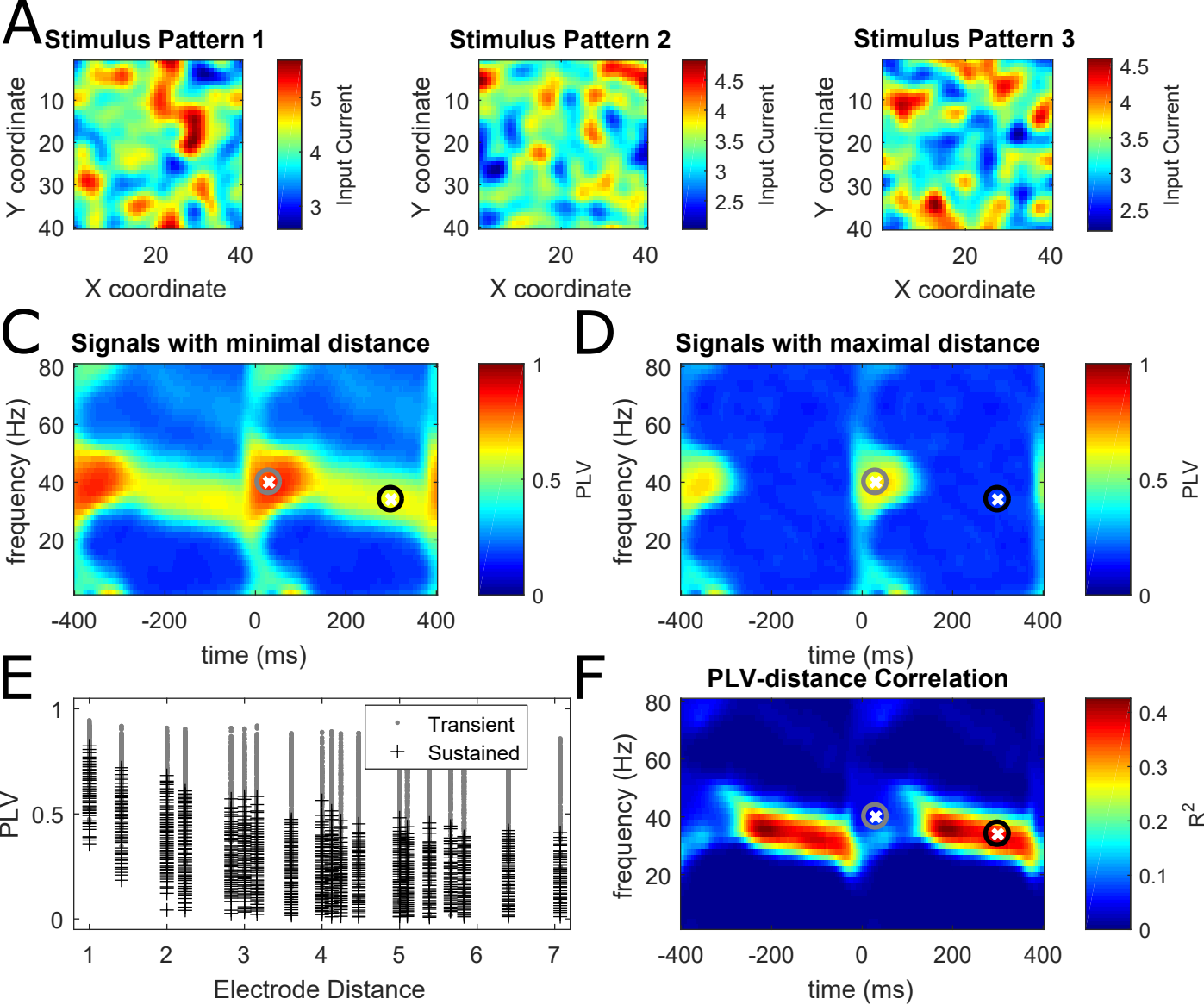

Supplement: S3 Fig — (A) During each interval between MSs, the network was presented with one of 3 possible stimuli. The stimulus patterns were randomly selected, but no 2 successive stimuli were the same. (C–F) As in S1 Fig. MS, microsaccade. (PDF) [file pbio.2004132.s003.pdf]

# A External phase-reset

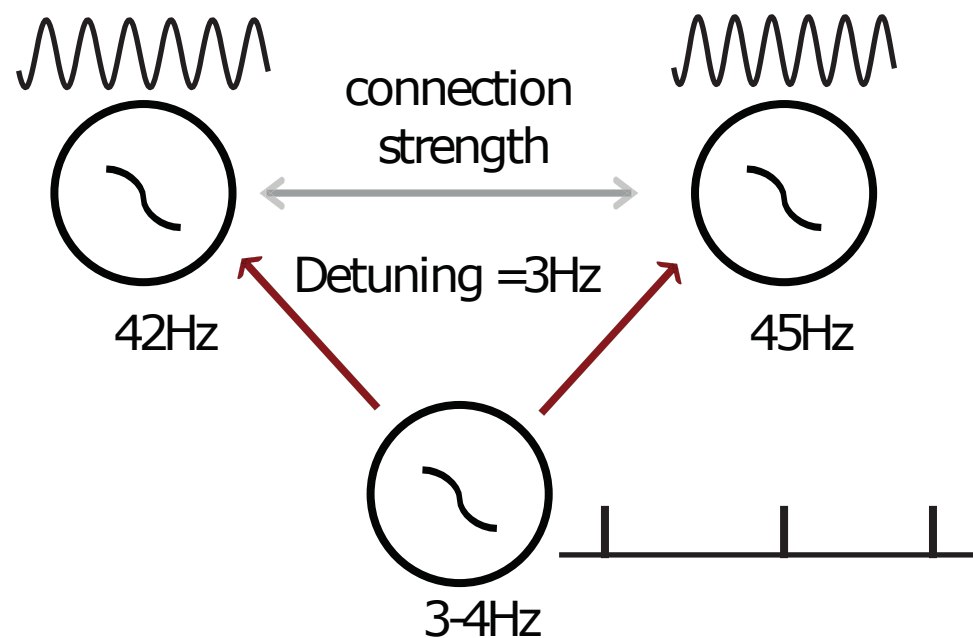

# B Mutual synchronization

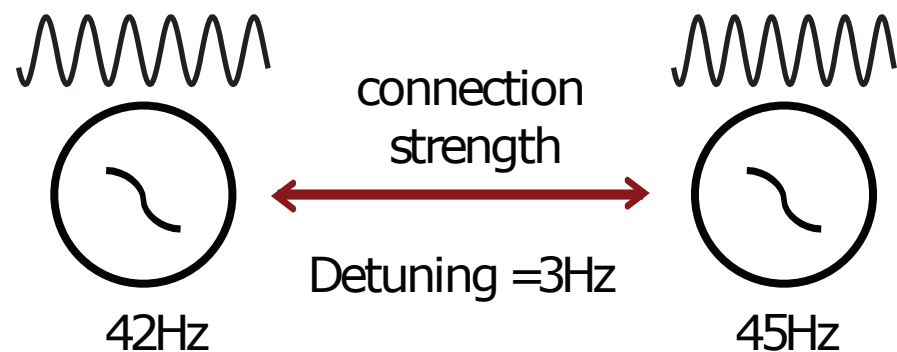

# C

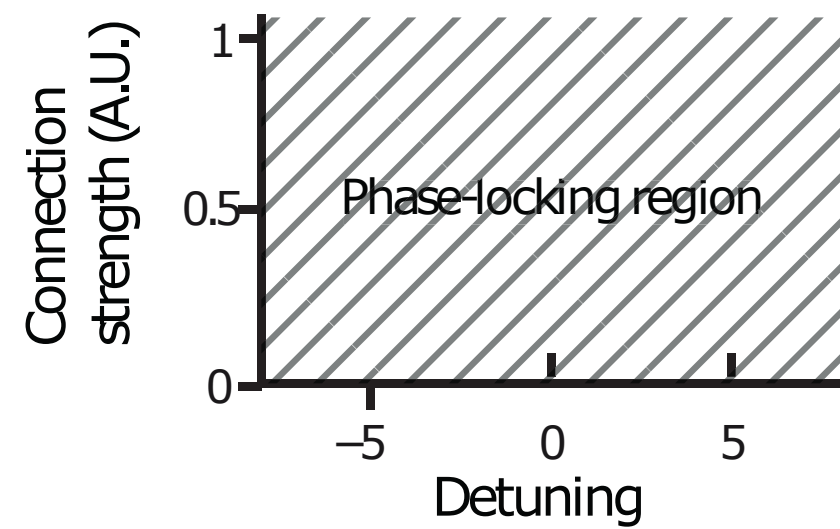

# D

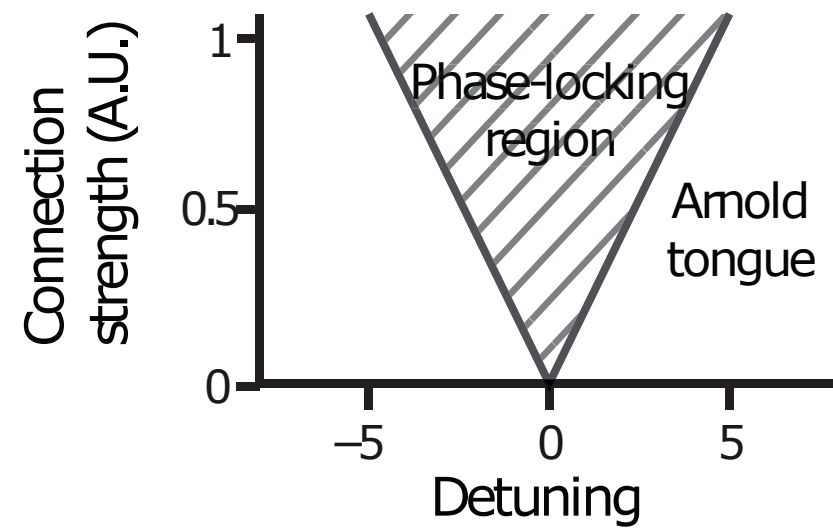

Supplement: S4 Fig — (A) Synchrony is achieved through (periodic) resetting by an outside source. (B) Synchrony arises through mutual interactions. (C) When synchrony is determined by an outside resetting pulse, connection strength and detuning no longer influence synchrony, therefore the Arnold Tongue is not visible in this case. (D) When synchrony is caused by mutual interactions, the connection strength has to be high enough to overcome any differences in intrinsic frequency (detuning). This results in a triangular region of synchrony also known as “the Arnold Tongue,” shown here. (PDF) [file pbio.2004132.s004.pdf]

A

Transient period

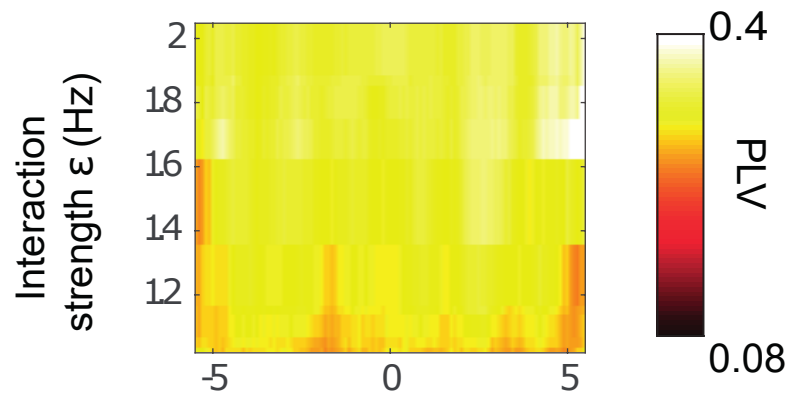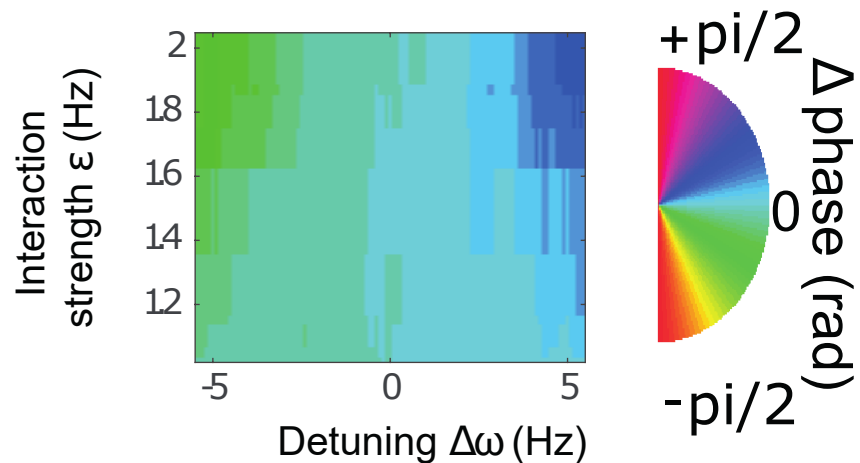

B

Sustained period

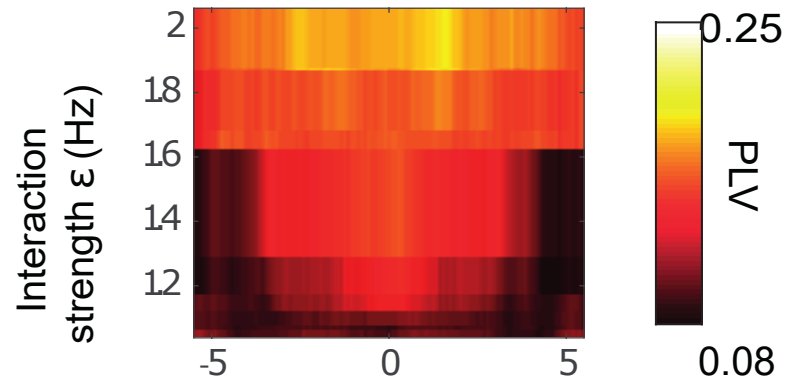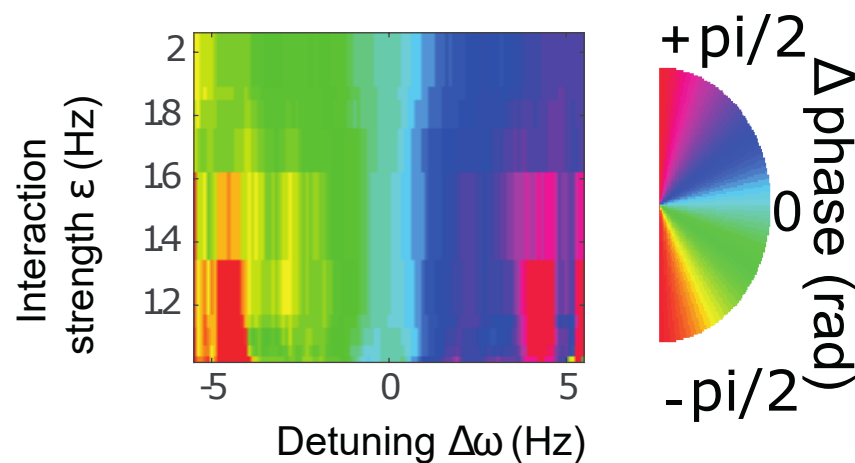

Supplement: S6 Fig — Based on the same dataset as the one used for Figs 4–6. Because connection strength and local input drive cannot be measured directly, interaction strength and detuning were used on the 2 axes (see above). LFP, local field potential. (PDF) [file pbio.2004132.s006.pdf]

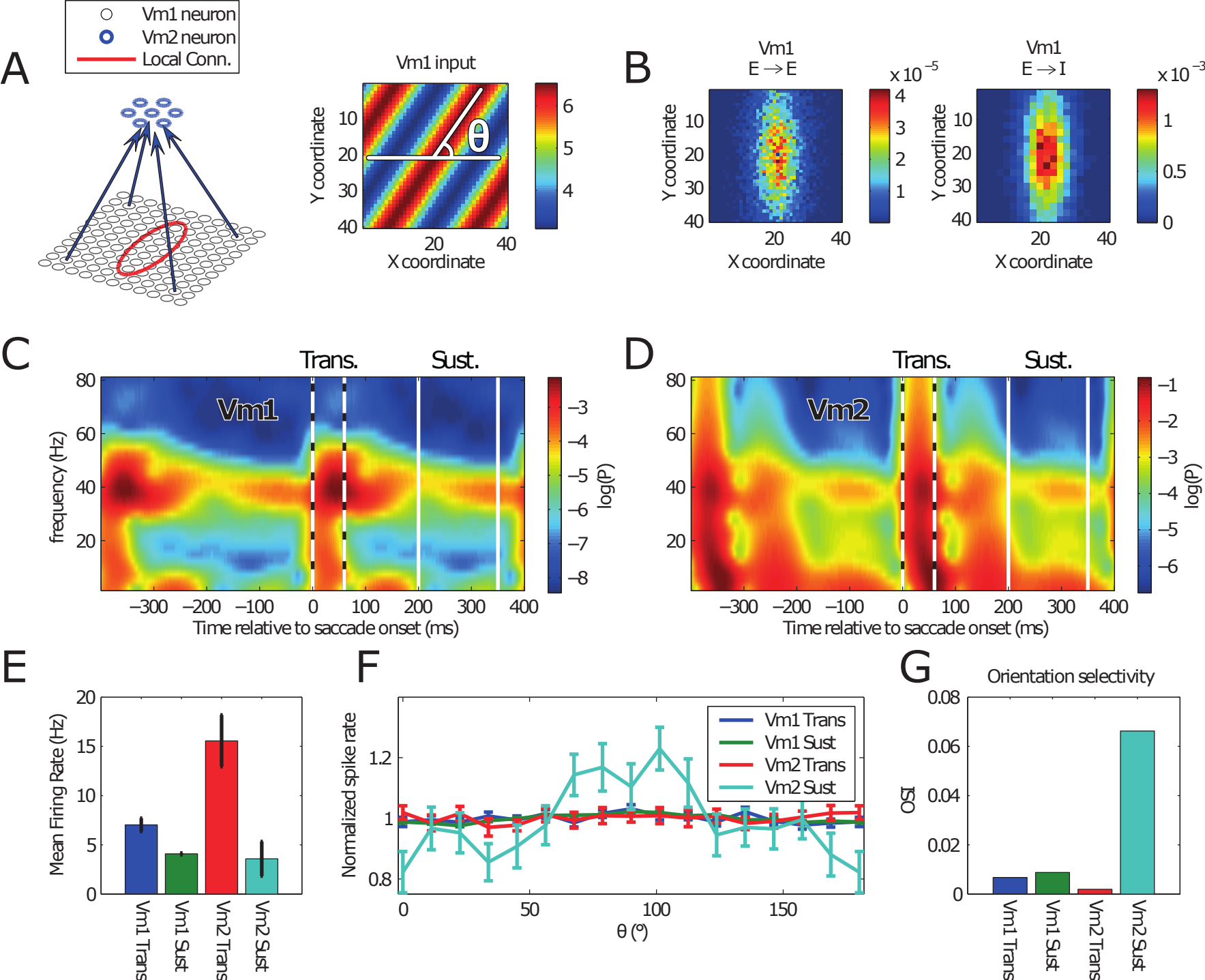

Supplement: S7 Fig — (A) Left: schematic representation of the network with its 2 subnetworks. Neurons in the first subnetwork (V1; 40 × 40 RSs, 20 × 20 FSs) receive direct input and project to the second subnetwork (V2; 100 RSs, 25 FSs) and to themselves. Neurons in V2 only project locally, i.e., there is no feedback to V1. The local connections in V1 were sampled from anisotropic Gaussians, illustrated by the red oval (see panel B). The feedforward connections (from V1 to V2) and local connections in V2 were sampled from a uniform distribution. Right: an example of the direct input to V1. V1 received one of 16 differently oriented gratings. The orientation (θ) of the grating is illustrated by the white overlay. (B) The connections from the center excitatory neuron in V1. Left: connections to other excitatory neurons within V1. Right: connections to inhibitory neurons within V1. (C, D) TFR of the mean LFP power, locked to saccade onset. Average across the electrodes in V1 (C) or for the single electrode in V2 (D). The vertical lines indicate the borders of the transient (0–70 ms, dashed) and the sustained period (200–350 ms, solid white) that were used for analysis in E–G. (E) The mean firing rates for the 2 response periods in V1 and V2. Error bars denote standard deviation across the 50 MSs. (F) The modulation of spike rate (normalized by the orientation average) as a function of stimulus orientation during the transient and sustained periods in V1 and V2. Error bars denote standard error of the mean across the 50 MSs. (G) Orientation sensitivity in panel E quantified by calculating the OSI (see S1 Text). FS, fast-spiking neuron; LFP, local field potential; MS, microsaccade; OSI, orientation selectivity index; RS, regular-spiking neuron; TFR, time-frequency representation. (PDF) [file pbio.2004132.s007.pdf]
