# Supplementary material for: Microsaccade-rhythmic modulation of neural synchronization and coding within and across cortical areas V1 and V2
Source: PLoS Biol. 2018 May 31;16(5):e2004132. doi: 10.1371/journal.pbio.2004132 (PMC5997357; doi:10.1371/journal.pbio.2004132)
Supplement: S5 Fig — The simulation used for this analysis contained a network with isotropically connected neurons (as in Fig 3 and panel A) with locally varying input strength. (A) In the transient period, coherence (quantified by the PLV; see Materials and methods) was high for any combination of connectivity strength and input difference (left), whereas in the sustained period, PLV was dependent on both input difference and connectivity (right). The synchronization region had a triangular shape known as the Arnold tongue. (B) The same as panel A, but for mean phase difference. PLV, phase-locking value. (PDF) [file pbio.2004132.s005.pdf]

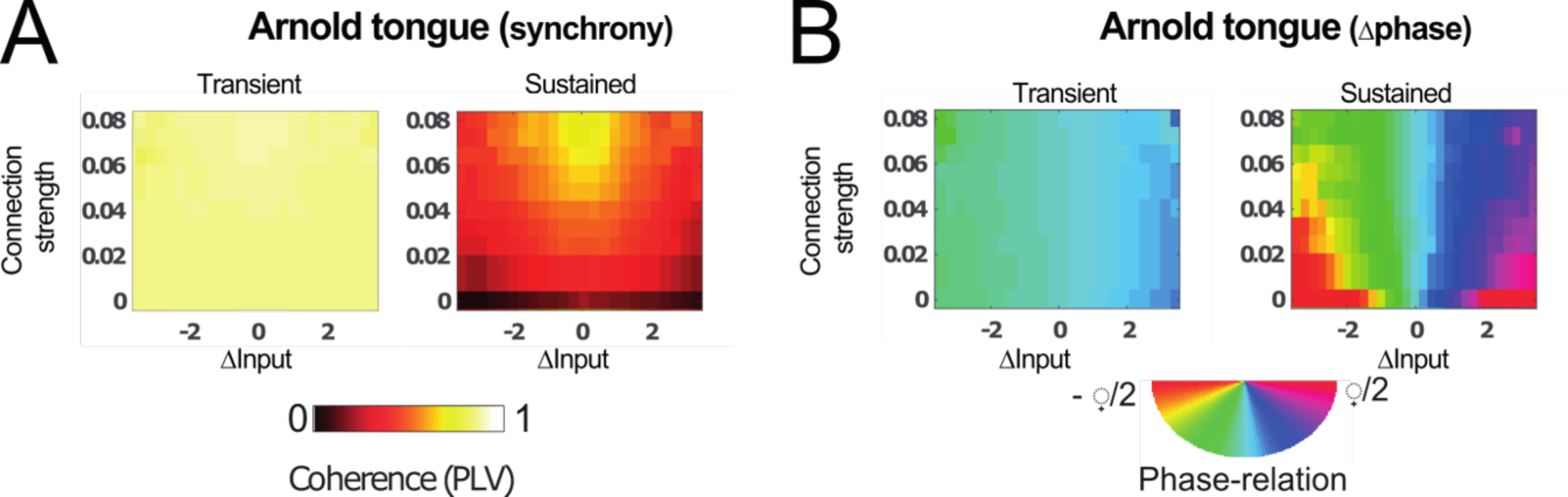
A
Arnold tongue (synchrony)
Transient
Sustained
Connection strength
0.08
0.06
0.04
0.02
0
-2
0
2
ΔInput
0
1
Coherence (PLV)
B
Arnold tongue (Δphase)
Transient
Sustained
ΔInput
Phase-relation
