# Supplementary material for: Microsaccade-rhythmic modulation of neural synchronization and coding within and across cortical areas V1 and V2
Source: PLoS Biol. 2018 May 31;16(5):e2004132. doi: 10.1371/journal.pbio.2004132 (PMC5997357; doi:10.1371/journal.pbio.2004132)
Supplement: S1 Text — (1) The effect of increasing MS interval time on the network model and on V1 LFP recording sites. (2) Simulations showing that MS-locked effects persist if stimulus changes after each MS. (3) Proposal of a general theoretical framework to understand MS-locked synchronization changes. (4) Discussion of the theory of weakly coupled theory and the Arnold tongue and demonstration that the Arnold tongue can only be reconstructed in the sustained part of the MS interval in the network model and between V1 recording sites. (5) Additional simulations to illustrate functional implications MS-locked synchronization changes. We show that stimulus orientation sensitivity of a network model differs between the 2 phases within the MS interval. (6) Supplementary methods. LFP, local field potential; MS, microsaccade. (DOCX) [file pbio.2004132.s008.docx]

# S1 Text

## Increasing the inter-saccade interval prolongs the sustained period

The analyses presented in Fig 2 were repeated for a simulation where the interval between MSs was lengthened (S1 Fig). The gamma-band synchrony was stable enough to last throughout the longer inster-saccade interval. For our experimental V1 data, we also repeated analysis to show MS-triggered TFR power for longer MS intervals (S2 Fig). Importantly, even with longer MS intervals, the sustained gamma-band remains dominant till the next MS.

## MS-locked effects persist across stimulus change

The simulated network Fig 2 was presented the same single stimulus over and over again for every saccade. In reality the stimulus on the retina will change between saccades, since a saccade consists of an eye movement. S3 Fig shows the result of a control simulation where the network was never presented with the same stimulus in successive saccades. The figure follows the conventions of Fig 2 and illustrates that the fact that the stimulus shown in previous inter-saccade interval is not of great importance (if at all) to the MS-locked gamma dynamics (i.e. the transient-sustained period dynamics).

## General view on temporal coordination of MS-shaped cortical activity

We can understand the differences in the transient activity shortly after the MS and the “sustained” period following it in the interval between MSs using general principles of synchronization theory [1–3].

We hypothesize that the phase-coordination of cortical activity during the transient and the sustained period rely on two distinct mechanisms. Phase-coordination in the transient period results from an external common resetting impulse (S4A Fig). Conversely, in the sustained period phase-coordination is achieved through mutual synchronization (S4B Fig).

A resetting impulse can either phase shift an ongoing oscillation or start the generation mechanism in the absence of an ongoing oscillation. Transient neural activation that travels from the retina along the visual hierarchy (feedforward sweep, [4]), as observed after stimulus onsets or (micro)saccades [5], can provide such global and external resetting impulses for a visual cortical area like V1. For MSs, a top-down corollary discharge originating from fronto-parietal regions as well as subcortical regions, might further shape the transient-related resetting (including pre-saccadic inhibition, [6]). Importantly, the external resetting impulses are independent of connectivity of V1 neurons. Furthermore, they act at a fast time-scale and large spatial-scale. However, the phase coordination is not sustainable and fades quickly after the end of the resetting impulse.

It is important to distinguish between the observed and the intrinsic frequency of an oscillator, when investigating phase-coordination through mutual synchronization. The intrinsic frequency is the frequency of the oscillator receiving input, if it were completely decoupled from all other oscillators. However, sufficiently coupled oscillators mutually adjust their phases (by phase delay/advance) over time such as to converge on a common frequency [3]. This will lead to an observed frequency differing from the intrinsic frequency. Two important variables regulate the synchronization process: The first factor is the interaction (coupling) strength. The interaction strength defines how strongly the phase of an oscillator is shifted (advanced or delayed) by another oscillator. In neural terms, interaction strength is mainly defined by anatomical connection strength between two oscillating (sub)networks and by the amplitude of an oscillation [7]. The second factor is detuning, defined as the difference in intrinsic frequency of the two oscillators. The larger the detuning, the stronger the coupling needs to be for the oscillators to converge to a common frequency. The most common in-vivo modulator of detuning is the variability of the network input. For gamma-band activity in V1, it has been shown that the excitatory input drive, determined largely by visual contrast, strongly modulates the intrinsic frequency in a retinotopic manner [8–11]. The regulation of synchronization by interaction strength and detuning is best visualized by mapping the synchronization values in the 2D space spanned by these two parameters [1–3] . This mapping yields a well-defined region of synchronization with an inverted triangular shape, the so-called Arnold tongue [12] (S4D Fig).

## Characterization of the Arnold Tongue

The approach to derive the so-called ‘Arnold tongue’ from the above-described empirical recording data (Fig 4) is described in detail in [13]. The Arnold tongue is a prediction about how the relationship between detuning (intrinsic frequency difference) and interaction strength (related to connectivity strength) determines the synchronization behavior among oscillators (or oscillating neural populations). In accordance to the theory of weakly coupled oscillators [1,2,14], oscillatory synchronization can be understood as resulting from a synchronization force acting through phase adjustments or delays pushing interacting oscillators towards sufficiently matching frequencies. Desynchronization (dephasing) emerges when oscillators have insufficiently matching frequencies. Two parameters critically determine the synchronization behavior: *detuning* and *interaction strength*.

**Detuning.** Oscillators have a preferred frequency at which they like to oscillate (also called natural or intrinsic frequency). If two oscillators have different preferred frequencies, we speak of ‘detuning’. If two oscillators do not interact, the detuning can be measured as the frequency difference between the two oscillators. However, if two oscillators interact and synchronize (hence reducing their frequency difference) the observed frequency difference will be generally smaller than their detuning. To empirically estimate ‘Detuning’ in simulation or experimental data, we used an approach that estimates the interaction function, which then allows to estimate the detuning. The method is outlined in detail in [13].

**Interaction strength.** Interaction strength is the amount of influence exerted between oscillators. More specifically, it is the amount of phase advance or delay that an oscillator has due to another oscillator. Whether it is phase advance or delay is defined by the interaction function. Phase advance or delay is by definition nothing else than an increase or decrease of the instantaneous frequency (rate of phase change over time) of an oscillator. The estimation of the interaction strength in simulation and experimental data is explained in detail in [13]. In short, it is quantified here as the amount of observed instantaneous frequency modulations that depend on the phase difference between two rhythms. In [13] it was shown that in neural network simulations, the interaction strength is closely related to synaptic connection strength, and that the interaction strength measure decreases monotonically with cortical distance in V1 as expected from horizontal connectivity spread.

**Arnold tongue**. The Arnold tongue is defined as the triangular synchronization region occurring in the parameter space of detuning and interaction strength (see S4 Fig, right hand panel). The triangular shape emerges, because the larger the interaction strength (higher y coordinate), the more detuning two oscillators can tolerate and still synchronize (larger span of high synchronization values in x-coordinates). We consider synchrony to be on the Arnold tongue when there is 1:1 phase locking between oscillators

As synchronization is sensitive to input-induced detuning (intrinsic frequency difference) and to local connectivity (interaction strength) only in the sustained period, we expected that the Arnold tongue would only be detectable in the sustained period.

To test this idea, we mapped the Arnold tongue in our network simulation under transient and sustained conditions. To that aim, we spatially varied the input strength in a network with isotropically connected neurons as shown previously (Fig 2 A,B). In this simulation, we placed a LFP electrode on every neuron and mapped all the PLV values between network locations during the transient as well as sustained period as a function of the neurons’ connection strength and input difference. We indeed only observed the Arnold tongue in the sustained period (S5A Fig).

In previous work [9], we have shown that within the Arnold tongue, i.e. the triangular region showing synchronization, frequency differences are translated into phase differences. We therefore predicted a stronger relationship between input difference and phase difference in the sustained period. To test this, we also mapped the phase difference between network location in the parameter space of connectivity strength and input difference (S5B Fig). As expected, we observed differences between the transient and sustained period after a MS. Specifically, although in both periods phase-differences coded for the input-difference, the phase-difference depended much more strongly on input difference in the sustained period. In addition, in the sustained period the phase difference depended on connectivity strength (i.e. the phase-relation profile changes for different connection strengths in the right panel of S5B Fig, but not in the left). This is expected from synchronization theory [1–3] and has been recently observed for the V1 gamma-band [13,15].

S6C/D Fig replicate the *Arnold Tongue* results from the model network in S5A/B Fig. Similar to our model, coherence in the V1 recordings was high in the transient period, and independent of local input differences (approximated by *detuning*), or connectivity (approximated by *interaction strength*)*.* On the other hand, gamma-band activity in the sustained period shows the triangular Arnold Tongue profile, in agreement with the theory of coupled oscillators.

## Supplementary methods

### Neural connectivity

For S7 Fig connectivity was similar to that of Fig 3. All connections in the x-direction were scaled with a factor of 3 before generating the connections profile, leading to the elliptical profiles seen in S7B Fig (this was done for both the excitatory and inhibitory connections, the latter are not shown in S7B Fig). The connection probabilities within V2 were all set to be uniform (similar to using very large values for $\sigma_{S}$ in equation 13). Feedforward connections between V1 and V2 were only excitatory and were uniform.

### Quantifying orientation sensitivity

We used the orientation selectivity index (OSI) defined as the mean vector length [16] in the complex plane to quantify orientation sensitivity in S7G Fig. It is defined as

1. $\text{ OSI=}\frac{\text{1}}{\sum_{\text{k}} \text{r}_{\text{k}}}\left| \sum_{\text{k}} \text{r}_{\text{k}}\exp\left( \text{i2}\text{θ}_{\text{k}} \right) \right|$

In this equation, $r_{k}$ is the activity in response to stimulus *k* (e.g. the sum of action potentials in V2 in a time window). Stimulus *k* has orientation of $\theta_{k}$ radians.

## Illustration of possible functional implications of the two temporal-coordination mechanisms for inter-areal processing and communication

Fig 3 showed that local connections can change the synchrony during the sustained period of the inter-saccade interval. To illustrate possible consequences of changes in synchrony for information processing, we conducted additional simulations with a network consisting of two visual model (sub-)networks (V1 and V2, S7A Fig). The first sub-network (V1) was a 40 × 40 PING network similar to the one shown in Fig 2A. V1 received direct input modulated by the MS kernel (Fig 1 A) consisting of a sinusoidal grating (S7A Fig, right). Similar to Fig 3B, left, we gave structure to the local connectivity in V1, sampling the connections from anisotropic Gaussian distributions. The resulting elliptical connectivity profiles (S7A,B Fig) made our model V1 sensitive to the orientation of an input pattern. This anisotropic connection pattern resembles the experimental finding of anisotropy in the long horizontal connections in the tree shrew striate cortex [17]. This connection pattern might facilitate processing of (the curvature of) line segments. The purpose of the model was not to capture the emergence of orientation tuning occurring in single cells at columnar level in V1. Instead, the model captured the effect of anisotropic (‘oriented’) spatial distribution of horizontal connections and their impact on spatial coordination of V1 output and receiving neurons in V2.

The second sub-network (V2) represented one column in visual cortex (higher in the visual hierarchy than V1) and consists of a collection of 100 excitatory and 25 inhibitory neurons that receive input from all V1 neurons and were interconnected uniformly (see Methods). In addition to the feed-forward drive from the V1 model, the V2 model also received a uniform background drive to increase the neurons’ excitability.

S7C/D Fig show the TFR of the power in V1 and V2 respectively. Both V1 and V2 generated the characteristic transient with high firing rate after the onset of the simulated saccade, followed by a sustained gamma response with lower firing rate (see S6E Fig for differences in firing rates between the transient and sustained phases).

S7F/G Fig illustrate the main point: a consequence of the different gamma synchronization dynamics in the sustained period compared to the transient. The V1 sub-network was presented with gratings of 16 different orientations (one of these shown in S7A Fig, right). S7F Fig shows that the average spike rate of V1 was independent of stimulus orientation during the whole intersaccade interval (resulting in a similar number of spikes sent to V2 for each orientation). The same holds for the transient period in V2. In the sustained period however, the anisotropic connectivity caused different synchrony in V1, which in turn altered the input to V2 (not in the number of spikes, but in the timing thereof). This caused the grating “parallel” to the connectivity (i.e. vertically oriented, similar to the connection patterns in panel B) to produce a stronger response in V2. This effect was quantified in S7G Fig by calculating the mean vector length (see Methods). In summary, the differences in gamma synchrony resulting from the (anisotropic) connection pattern in V1 made it possible for V2 to detect oriented spatial arrangements of stimuli during the sustained period, whereas this was not possible during the transient. Note that the transient activity in V1 still reflected the individual neuron input, and therefore permitted some sort of rate or latency code (see also the left panel of Fig 3D). However, due to the uniform connectivity from V1 to V2, the V2 network was not sensitive to such details.

In Fig S7, our model displayed some sort of orientation preference that is not entirely congruent with established models for the emergence of orientation selectivity [18,19]. Indeed, our model V1 units were insensitive for orientation, selectivity only emerges in V2 as a consequence of connectivity within V1. In reality, neurons in V1 [20], LGN [19,21] and even in the retina [22] have been shown to exhibit orientation selectivity. Therefore, the results in S7 Fig should not be interpreted as a good model for orientation selectivity in striate cortex, but rather an example of how horizontal connections and synchrony can influence network processing in the sustained period. Note, however, that orientation selective cells have been shown to preferentially project along one axis in retinotopic coordinates not unlike the connection pattern in S7A Fig [17]. Effects like those in S7 Fig can conceivably contribute to emergence of orientation selectivity along the visual pathway [19]. Our model results predict that this effect may change orientation tuning down-stream of area V1 in the sustained period compared to the transient.

1. Kopell N, Ermentrout GB. Chapter 1 Mechanisms of phase-locking and frequency control in pairs of coupled neural oscillators. Handb Dyn Syst. 2002;2: 3–54. doi:10.1016/S1874-575X(02)80022-4

2. Izhikevich EM. Dynamical Systems in Neuroscience: The Geometry of Excitability and Bursting [Internet]. Dynamical Systems. 2007. doi:10.1017/S0143385704000173

3. Pikovsky A, Rosenblum M, Kurths J, Hilborn RC. Synchronization: A Universal Concept in Nonlinear Science. Am J Phys. 2002;70: 655. doi:10.1119/1.1475332

4. Lamme VAF, Roelfsema PR. The distinct modes of vision offered by feedforward and recurrent processing. Trends in Neurosciences. 2000. pp. 571–579. doi:10.1016/S0166-2236(00)01657-X

5. Rajkai C, Lakatos P, Chen C-M, Pincze Z, Karmos G, Schroeder CE. Transient cortical excitation at the onset of visual fixation. Cereb Cortex. 2008;18: 200–209. doi:10.1093/cercor/bhm046

6. Reppas JB, Usrey WM, Reid RC. Saccadic Eye Movements Modulate Visual Responses in the Lateral Geniculate Nucleus. Neuron. 2002;35: 961–974. doi:10.1016/S0896-6273(02)00823-1

7. Womelsdorf T, Schoffelen J-M, Oostenveld R, Singer W, Desimone R, Engel AK, et al. Modulation of neuronal interactions through neuronal synchronization. Science. 2007;316: 1609–1612. doi:10.1126/science.1139597

8. Jia X, Xing D, Kohn A. No consistent relationship between gamma power and peak frequency in macaque primary visual cortex. J Neurosci. 2013;33: 17–25. doi:10.1523/JNEUROSCI.1687-12.2013

9. Lowet E, Roberts M, Hadjipapas A, Peter A, van der Eerden J, De Weerd P. Input-Dependent Frequency Modulation of Cortical Gamma Oscillations Shapes Spatial Synchronization and Enables Phase Coding. PLoS Comput Biol. 2015;11: e1004072. doi:10.1371/journal.pcbi.1004072

10. Ray S, Maunsell JHR. Differences in gamma frequencies across visual cortex restrict their possible use in computation. Neuron. Elsevier Inc.; 2010;67: 885–96. doi:10.1016/j.neuron.2010.08.004

11. Roberts MJ, Lowet E, Brunet NM, Ter Wal M, Tiesinga P, Fries P, et al. Robust gamma coherence between macaque V1 and V2 by dynamic frequency matching. Neuron. Elsevier Inc.; 2013;78: 523–36. doi:10.1016/j.neuron.2013.03.003

12. Boyland PL. Bifurcations of circle maps: Arnol’d tongues, bistability and rotation intervals. Commun Math Phys. 1986;106: 353–381. doi:10.1007/BF01207252

13. Lowet E, Roberts MJ, Peter A, Gips B, de Weerd P. A quantitative theory of gamma synchronization in macaque V1. Elife. eLife Sciences Publications Limited; 2017;6: e26642. doi:10.7554/eLife.26642

14. Schwemmer MA, Lewis TJ. Phase Response Curves in Neuroscience. Phase Response Curves in Neuroscience. 2012. pp. 3–31. doi:10.1007/978-1-4614-0739-3

15. Vinck M, Lima B, Womelsdorf T, Oostenveld R, Singer W, Neuenschwander S, et al. Gamma-phase shifting in awake monkey visual cortex. J Neurosci. 2010;30: 1250–1257. doi:10.1523/JNEUROSCI.1623-09.2010

16. Ringach DL, Shapley RM, Hawken MJ. Orientation selectivity in macaque V1: diversity and laminar dependence. J Neurosci. 2002;22: 5639–51. doi:20026567

17. Bosking WH, Zhang Y, Schofield B, Fitzpatrick D. Orientation Selectivity and the Arrangement of Horizontal Connections in Tree Shrew Striate Cortex. J Neurosci. 1997;17: 2112–2127. Available: http://www.jneurosci.org/content/17/6/2112.short

18. Somers DC, Nelson SB, Sur M. An emergent model of orientation selectivity in cat visual cortical simple cells. J Neurosci. 1995;15: 5448–5465.

19. Scholl B, Tan AYY, Corey J, Priebe NJ. Emergence of Orientation Selectivity in the Mammalian Visual Pathway. J Neurosci. 2013;33: 10616–10624. doi:10.1523/JNEUROSCI.0404-13.2013

20. Hubel D, Wiesel T. Receptive fields, binocular interaction and functional architecture in the cat’s visual cortex. J Physiol. 1962;160: 106–54.

21. Zhao X, Chen H, Liu X, Cang J. Orientation-selective responses in the mouse lateral geniculate nucleus. J Neurosci. 2013;33: 12751–63. doi:10.1523/JNEUROSCI.0095-13.2013

22. Antinucci P, Abbas F, Hunter PR. Orientation Selectivity in the Retina: ON Cell Types and Mechanisms. J Neurosci. 2016;36: 8064–6. doi:10.1523/JNEUROSCI.1527-16.2016
